# Diverse sensitivities of TRPA1 from different mosquito species to thermal and chemical stimuli

**DOI:** 10.1038/s41598-019-56639-w

**Published:** 2019-12-27

**Authors:** Tianbang Li, Claire T. Saito, Tomoyuki Hikitsuchi, Yoshihiro Inoguchi, Honami Mitsuishi, Shigeru Saito, Makoto Tominaga

**Affiliations:** 1 Department of Physiological Sciences, SOKENDAI, Okazaki, Japan; 2 Division of Cell Signaling, National Institute for Physiological Sciences, Okazaki, Japan; 3 Thermal Biology Group, Exploratory Research Center on Life and Living Systems (ExCELLS), Okazaki, Japan; 4 Research & Development Laboratory, Dainihon Jochugiku Co., Ltd., Osaka, Japan

**Keywords:** Cell biology, Physiology

## Abstract

Temperature and odors profoundly affect the behavior of animals. Transient receptor potential channel, subfamily A, member 1 (TRPA1) functions as a polymodal nociceptor for sensing both vital environmental cues in insects. Mosquitoes are recognized as disease vectors, and many efforts have been devoted to investigations of their host-seeking behaviors and repellents. However, the physiological characteristics of mosquito TRPA1 have not been systematically studied. We identified multiple alternative splice variants of the *TrpA1* gene from *Anopheles gambiae*, *Anopheles stephensi*, *Aedes aegypti* and *Culex pipiens pallens* mosquitoes. And we performed comparative analyses of the responses of mosquito TRPA1s to heat or chemical stimuli with calcium-imaging and whole-cell patch-clamp methods. Comparison of TRPA1 among four mosquito species from different thermal niches revealed that TRPA1 of *Culex pipiens pallens* inhabiting the temperate zone had a lower temperature threshold for heat-evoked activation, which was supported by the *in vivo* heat-avoidance test. Notably, the chemosensitivity of mosquito TRPA1 channels revealed differences not only between variants but also among species. Moreover, we discovered three novel mosquito TRPA1 agonists. Thermal niches selection and evolutionary trajectories significantly affect the functional properties of mosquito TRPA1, which represents a hallmark of the behaviors that may permit the design of improved mosquito control methods.

## Introduction

Transient receptor potential (TRP) ion channels play important roles in various sensations including vision, olfaction, audition, gustation, thermosensation and mechanosensation^1–3^, guiding animals to use appropriate behaviors. Among them, TRP ankyrin 1 (TRPA1) is a calcium-permeable non-selective cation channel with multiple ankyrin repeats in the amino-terminus^4,5^. It functions as a polymodal receptor for detecting noxious temperature and adverse chemicals, suggesting that it is involved in nociception^6–8^. In insects, expression of TRPA1 was found in olfactory and gustatory receptor neurons as well as in the brain^9,10^.

During evolution animals adapt to habitats with different environmental factors, and even closely related species possess distinct optimal temperature ranges for survival^11–14^. Mosquitoes are crucial vectors for transmission of epidemic diseases in tropical and subtropical areas. And mosquito TRPA1 has been reported to be a critical nociceptor for sensing overheated ambient temperature and hazardous compounds^9,10,15^. However, most of the mosquito TRPA1 researches have been limited to *Anopheles gambiae*, the notorious malaria vector in West Africa^9,10,15,16^, and properties of TRPA1 channels of *Anopheles stephensi*, *Aedes aegypti* and *Culex pipiens pallens* are poorly characterized. Genus *Anopheles* and Aedes/Culex diverged approximately 150 million years ago. Moreover, these mosquitoe species inhabit different areas of the world, suggesting that mosquito TRPA1 channels might exhibit species-specific differences in sensitivity to both thermal and chemical stimuli.

Alternative splicing of pre-mRNA leads to generate different proteins encoded by the same gene^17^, a process that has been reported in vertebrates and invertebrates^10,18,19^. *Drosophila melanogaster’s* TRPA1 has multiple isoforms due to alternative splicing^18^, and they were reported to be expressed in a tissue-specific fashion^10^. The protein structures of these variants differ in the N-terminus as well as the linker region between the ankyrin repeat domain and transmembrane domain. Previously, different N-termini of TRPA1 variants of *Anopheles gambiae* were investigated and the studies revealed that those different structures are involved in distinct thermosensitivities and chemosensitivities^10,15,20^. However, whether this phenomenon is consistent in other disease vector mosquitoes remains unanswered.

In the present study, we identified and compared multiple variants of TRPA1 from 4 mosquito species. The channel properties were electrophysiologically characterized not only among mosquito species but also between alternative splicing variants. We discovered that temperature thresholds for heat-evoked activation of TRPA1 of mosquitoes could be indicative of their respective thermotaxis behaviors *in vivo*. We also found that phylogeny of mosquito species reflects the pattern of divergence in TRPA1 chemosensitivity. Understanding of the physiological functions of disease vector mosquito TRPA1 could be extended to shed light upon the mechanism by which TRPA1 functions in the regulation of mosquito host-seeking and avoidance behaviors.

## Results

### Cloning and identification of *TrpA1* variants from *Anopheles stephensi, Aedes aegypti* and *Culex pipiens pallens*

**Figure 1:**
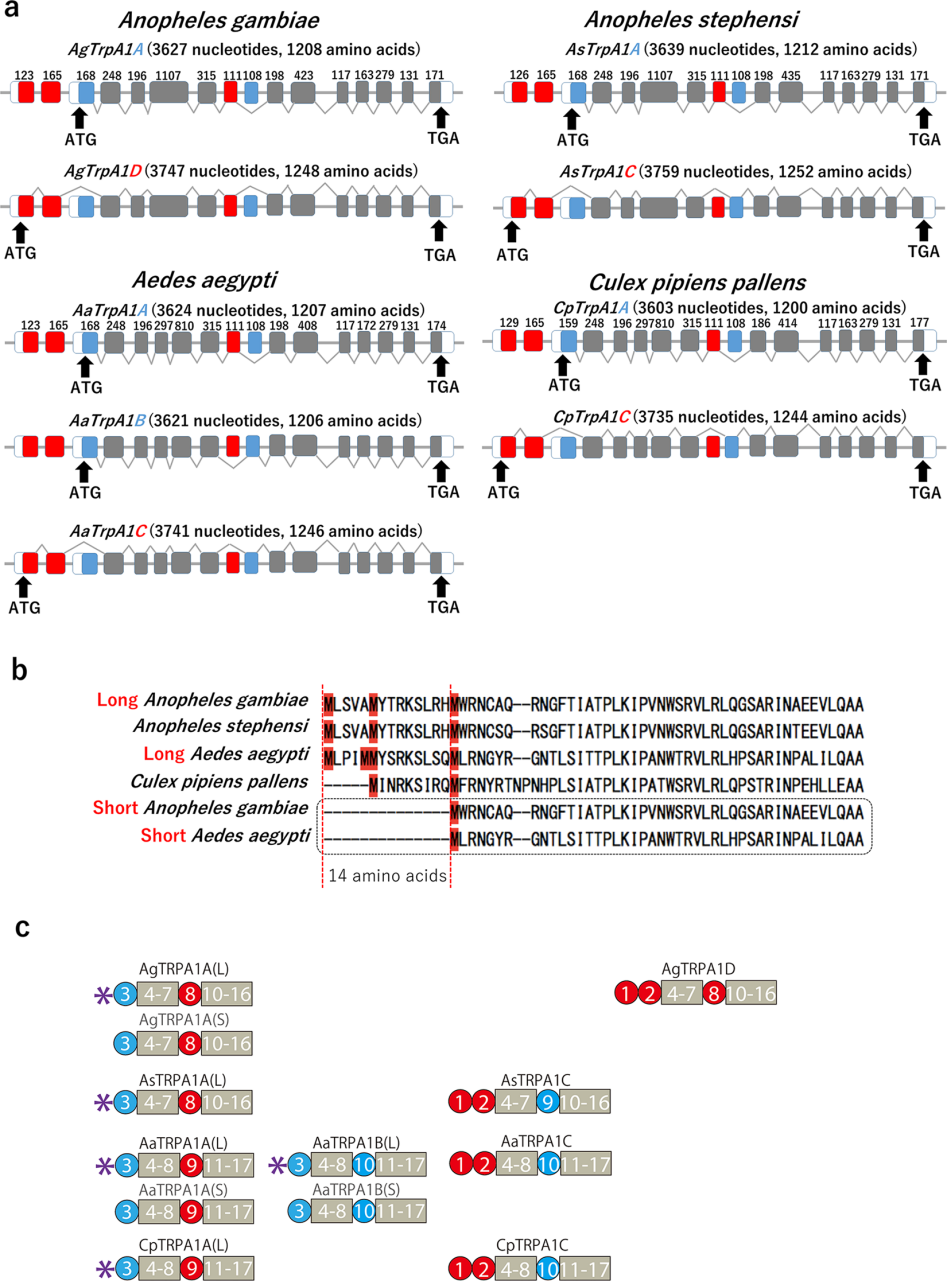
Multiple isoforms of TRPA1 are expressed by *Anopheles gambiae, Anopheles stephensi, Aedes aegypti* and *Culex pipiens pallens*. (**a**) Schematic mRNA structures of AgTrpA1A, AgTrpA1D, AsTrpA1A, AsTrpA1C, AaTrpA1A, AaTrpA1B, AaTrpA1C, CpTrpA1A and CpTrpA1C. Each rectangle represents an exon. Black arrows indicate the start point for translation. Exons in the same transcript are connected with grey broken lines. Blue and red boxes are alternatively spliced exons. (**b**) Amino acid sequence alignments of the N-terminus of AgTRPA1A, AaTRPA1A and AaTRPA1B. The black dot box indicates the previously reported short sequences of AgTRPA1A, AaTRPA1A and AaTRPA1B, which lose 14 amino acids (between 2 red dashed lines) compared with the long TRPA1B sequences. Methionine codons located in this region are highlighted in red. (**c**) A cartoon summarizes all the mosquito TRPA1 transcripts which are systematically compared in the following experiments. Transcripts with (S) have been reported. Purple asterisks indicate the peptides which were newly recognized in this study. Numbers in circles and boxes indicate the order of exons illustrated in (**a**).

To identify the splicing variants of *TrpA1* genes, 5′- and 3′-rapid amplification of cDNA ends (RACE) was performed to identify the upstream and downstream exons coding proteins. As a result, several different splicing variants were identified (Fig. 1a). Alternative splicing occurred in two different positions near the 5′ end and in the middle of *TrpA1* genes. The nomenclature in the present study refers to a previous study in regard to *Drosophila melanogaster* TRPA1^18^. *TrpA1* was newly cloned from *Anopheles stephensi* (As), *Aedes aegypti* (Aa) and *Culex pipiens pallens* (Cp) in the present study. Two kinds of splicing variants were identified from *Anopheles stephensi* and *Culex pipiens pallens* (*TrpA1A* and *TrpA1C*). Moreover, 3 kinds of splicing variants were identified from *Aedes aegypti* (*TrpA1A*, *TrpA1B*, and *TrpA1C*) (Fig. 1a).

In the course of the cloning work, we found that the N-terminal regions of the previously reported *Anopheles gambiae* TRPA1A (AgTRPA1A), AaTRPA1A and AaTRPA1B proteins were truncated. As illustrated in Fig. 1b, methionines were found 14 amino acids upstream of previously reported methionine by Kang *et al*. in *Anopheles gambiae* and *Aedes aegypti*^10^. A methionine was also found 9 amino acids upstream of the previously reported methionine in *Culex pipiens pallens*. Notably, these newly found initiation codons are located upstream of one previously reported in the same reading frame; and these methionine codons are also in the vicinity of the upstream termination codons (Fig. S1a,b). In addition, newly identified portions were well conserved among the 4 species. Thus, it is highly possible that these additional amino acids exist in the mosquito TRPA1 (Figs. 1b and S1a) and TRPA1 constructs previously cloned lack the 14 or 9 amino acids at the N-termini. Therefore, truncated AgTRPA1A was designated as AgTRPA1A(S), and AgTRPA1A with the newly identified amino acids in the N-terminus was designated as AgTRPA1A(L) when comparing these 2 different clones in later sections. Otherwise, we designated AgTRPA1A as AgTRPA1A(L) when it was used in electrophysiological experiments. A cartoon describes all the TRPA1 transcripts involved in this investigation (Fig. 1c). We chose some TRPA1 transcripts from Fig. 1c depending on purpose of the specific experiments (Fig. S1c).

### The rate of temperature rise influenced TRPA1 current density, but not temperature threshold for heat activation

Firstly, in order to establish a standardized method of *in vitro* recording to reduce experimental biases, we examined whether the speed of the heat-ramp affected the thermal responses of mosquito TRPA1, including activity (the current density) and sensitivity (temperature threshold for activation). AgTRPA1A(S) was initially exploited for this purpose since it was reported to elicit large enough current amplitudes by heat stimulation in a previous study^10^. AgTRPA1A(S) was transiently expressed in HEK293 cells and whole-cell ionic currents against heat stimulation were measured (Fig. S1c). The temperature of the bath solution was cooled down to about 15 °C prior to heat stimulation. Its temperature threshold for heat activation was generally measured near 30 °C based on an Arrhenius plot (Fig. S2a, right). The rates of heat-ramps were changed, and the current density and temperature threshold for activation were calculated from the heat-evoked ionic currents obtained from AgTRPA1A(S) expressed in HEK293 cells. The rate was calculated by measuring the time for a linear 10 °C temperature rise (Fig. S2a). Fig. S2b shows that the current density of AgTRPA1A(S) activated by heat stimulation was increased with the rate of temperature elevation, whereas the temperature threshold for activation was not affected by the change in rate of temperature elevation. These observations clearly indicated that the heat-ramp rate should be constant throughout the investigation. Therefore, all the heat-stimulation experiments in this study followed a protocol in which the rate of temperature increase was approximately 1.5 °C/sec.

### Addition of 14 amino acids to the TRPA1B N-terminus attenuated the channel activity

**Figure 2:**
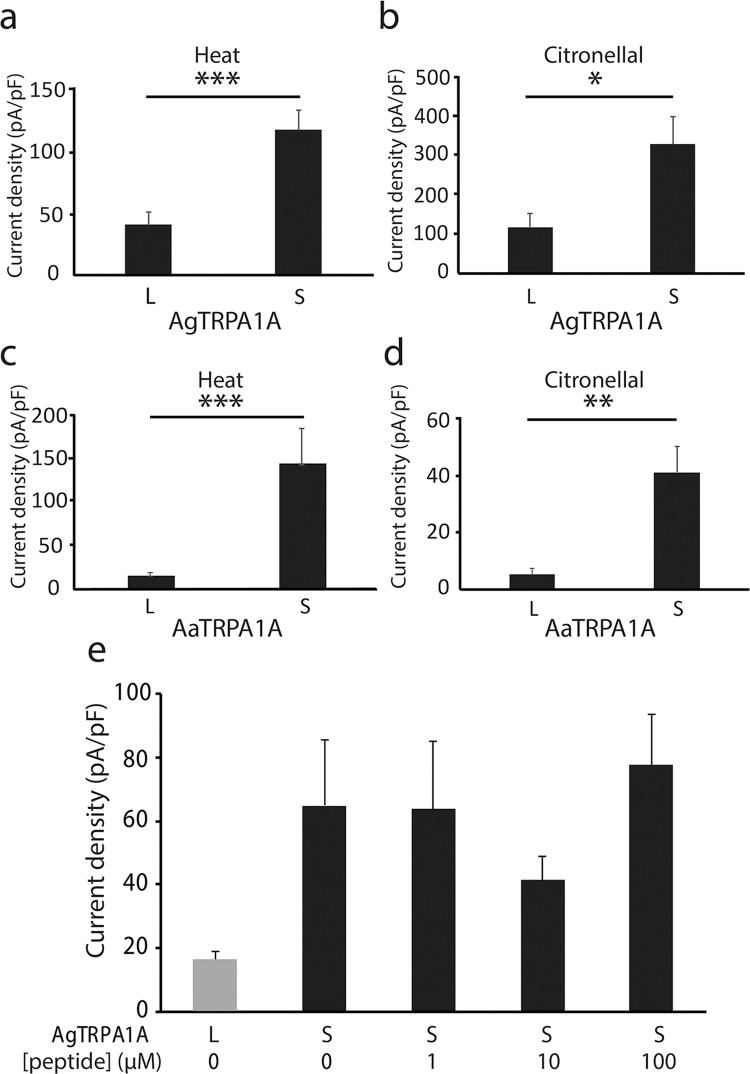
Addition of 14 amino acids to the N-terminus attenuated channel activity. (**a**,**b**) Density comparison of the heat-evoked currents (**a**) and 5 mM citronellal-evoked currents (**b**) observed in AgTRPA1A(S) and AgTRPA1A(L) expressed in HEK293 cells, respectively. (**c**,**d**) Density comparison of the heat-induced currents (**c**) and 3 mM citronellal-evoked currents (**d**) in AaTRPA1A(S) and AaTRPA1A(L) expressed by HEK293 cells, respectively. For (**a**–**d**), all values are means ± S.E.M; n ≥ 5; *P < 0.05; **P < 0.01; ***P < 0.001, unpaired Student’s *t*-test. (**e**) Comparison of the heat-evoked currents of AgTRPA1A(L) and AgTRPA1A(S) expressed in HEK293 cells with different intracellular concentrations of dissociative 14-amino acid-long peptide. All values are means ± S.E.M; n ≥ 8. No significant difference, one-way ANOVA with Bonferroni’s *post hoc* analysis.

It appeared that AgTRPA1D and AgTRPA1A showed distinct channel sensitivities to heat stimulus^10^. We hypothesized that the differences in sensitivities might be due to differences in the N-termini and that even very subtle difference in this region might determine specific channel properties observed in AgTRPA1A, AaTRPA1A and AaTRPA1B channels (with or without a short peptide at the N-terminus). Therefore, we investigated whether the additional 14 amino acids in the N-termini of AgTRPA1A and AaTRPA1A play an important role in modulating channel properties (Figs. 1b and S1c). Current densities of long and short AgTRPA1A (or AaTRPA1A) in response to heat stimuli or high concentrations of citronellal, a known chemical agonist, were compared in *Anopheles gambiae* (Fig. 2a,b) and *Aedes aegypti* (Fig. 2c,d), respectively. Surprisingly, the addition of 14 amino acids to the N-terminus of AgTRPA1A (or AaTRPA1A) significantly attenuated the density of heat- and citronellal-evoked currents in both mosquito species. Subsequently, the mechanism by which such a short peptide could affect the channel activity of TRPA1 was investigated by directly introducing the 14-amino acid-long peptide to the pipette solution. Densities of heat-evoked AgTRPA1A(S) currents were not changed by the added peptide at different concentrations (Fig. 2e). That observation ruled out the possibility that smaller currents evoked by heat in the cells expressing AgTRPA1A(L) were not caused by the binding of the 14 amino acids to the channel.

### A short alternative splicing exon between ankyrin repeats and the transmembrane domain is not important for AaTRPA1 temperature sensing

Among 4 mosquito species, TRPA1B isoform was solely identified in *Aedes aegypti* (Fig. 1a,c). The difference between a middle region in the transcript of AaTRPA1A (111 nucleotides) and that of AaTRPA1B (108 nucleotides) is caused by distinct exons in the intracellular region located between the last ankyrin repeat and the first transmembrane segment (Figs. 1a and S1b,c). This structure resembles the alternative splicing variants of TRPA1 found in *Drosophila melanogaster*. In *Drosophila*, this intracellular region has been reported to be critical for TRPA1 responses to heat stimuli^18^. Therefore, the significance of a single alternative splicing exon of *Aedes aegypti* TRPA1 (TRPA1A and TRPA1B) to heat stimulation was examined. The two channels comparably responded to heat stimulation in both the calcium-imaging experiments with Fura-2 (Fig. S3a) and the patch-clamp recordings (Fig. S3b). Hence, the amino acids located near the first transmembrane domain might not appear to have significant roles regarding the activity of TRPA1 in *Aedes aegypti*.

### Splicing variants of mosquito TRPA1 showed distinct thermal responses

**Figure 3:**
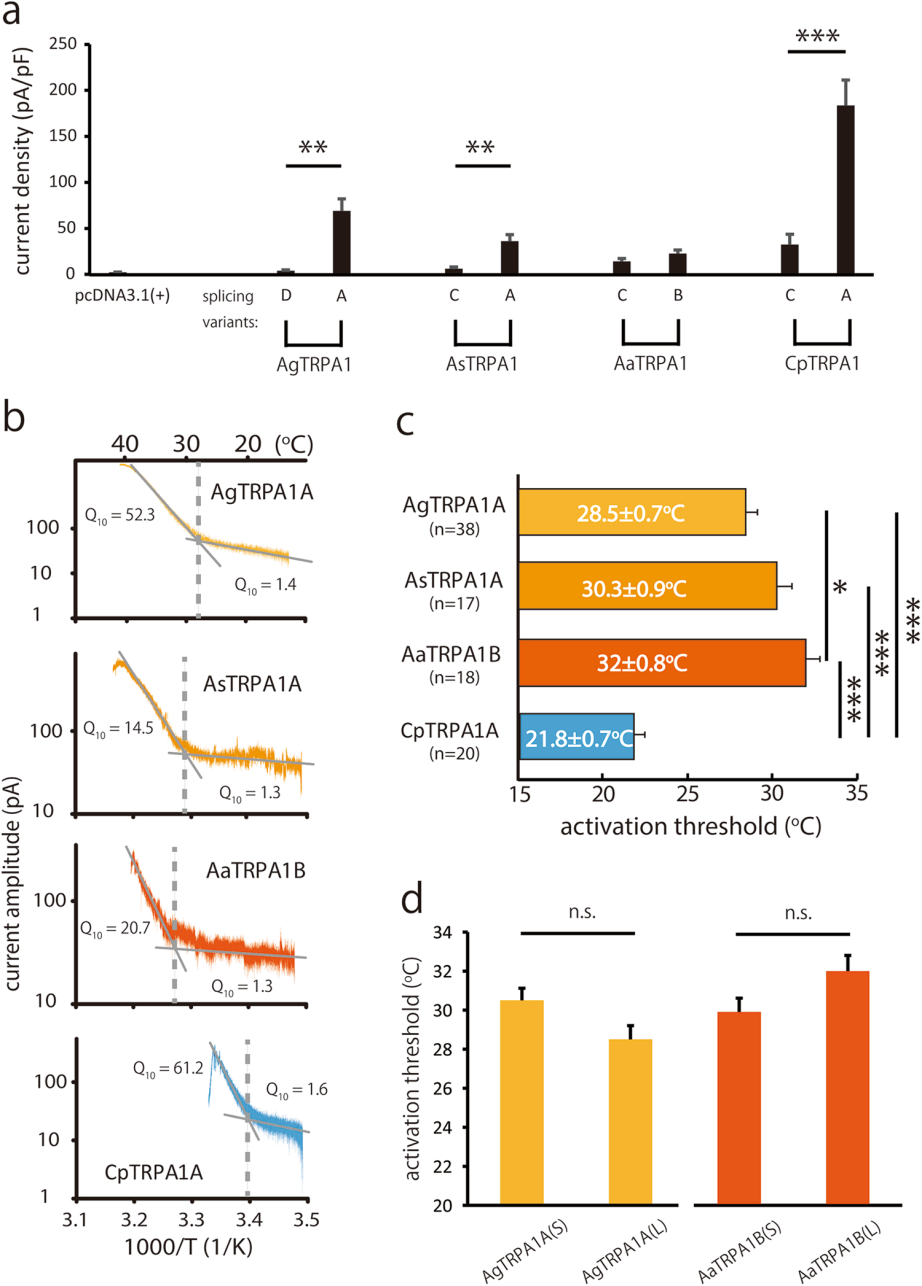
Divergent thermosensitivities of TRPA1 variants among different mosquito species. (**a**) Density comparison of the currents activated by increasing bath solution temperature from 15 °C to near 42 °C in HEK293 cells expressing AgTRPA1D, AgTRPA1A, AsTRPA1C, AsTRPA1A, AaTRPA1C, AaTRPA1B, CpTRPA1C or CpTRPA1A, **P < 0.01; ***P < 0.001, unpaired Student’s *t*-test. (**b**) Representative Arrhenius plots of the currents of AgTRPA1A, AsTRPA1A, AaTRPA1B and CpTRPA1A evoked by heat stimulation, from top to bottom, respectively. Dash lines indicate the temperature thresholds for heat-evoked activation. The average Q_10_ values higher and lower of the temperature thresholds were calculated and shown in the corresponding representative Arrhenius plots. (**c**) Temperature thresholds for heat-evoked activation are shown as means ± S.E.M. 10 ≤ n ≤ 60 each; **P < 0.01; ***P < 0.001, one-way ANOVA with Bonferroni’s *post hoc* analysis. (**d**) Comparison of the temperature thresholds for heat- evoked activation between AgTRPA1A(L) and AgTRPA1A(S) (left); between AaTRPA1B(L) and AgTRPA1A(S) (right). All values are means ± S.E.M; n ≥ 14. ns; unpaired Student’s *t*-test.

After excluding the effect of the intracellular region described above on thermosensitivity, the significance of the N-terminal difference was investigated within each species. TRPA1A showed significantly larger heat-evoked currents than did TRPA1D in *Anopheles gambiae*, TRPA1C in *Anopheles stephensi* and *Culex pipiens pallens* while the significant difference was not observed in *Aedes aegypti* (Figs. 3a and S1c). Moreover, temperature thresholds for heat-evoked activation of TRPA1A (or TRPA1B) of tropical mosquito species (*Anopheles gambiae*, *Anopheles stephensi* and *Aedes aegypti*) calculated with Arrhenius Plots (Fig. 3b) were significantly higher than that of TRPA1A from *Culex pipiens pallens* in Japan (a temperate zone) (28.5 ± 0.7, 30.3 ± 0.9, 32 ± 0.8 and 21.8 ± 0.7 °C for TRPA1 from *Anopheles gambiae*, *Anopheles stephensi*, *Aedes aegypti* and *Culex pipiens pallens*, respectively) (Figs. 3c and S4b). Whereas temperature thresholds for the N-terminus-truncated shorter form of TRPA1A (or TRPA1B) described in Fig. 2 showed no significant difference from their longer counterparts (Fig. 3d). Moreover, temperature coefficient (Q_10_) values calculated based on the flex points in the Arrhenius plots were slightly over 1 at temperatures below the thresholds, while they increased to huge values (over 14) above the thresholds (Fig. 3b). Temperature thresholds for heat-evoked activation of TRPA1A (or TRPA1B) showed the similar relationship with the geographic distribution of mosquito species (Figs. 3b and S4b). Thus, these data suggested that the thermosensitivity of mosquito TRPA1 channels was related to their distinct thermal tolerances.

### The temperature preference of mosquitoes reflected the thermosensitivity of mosquito TRPA1

**Figure 4:**
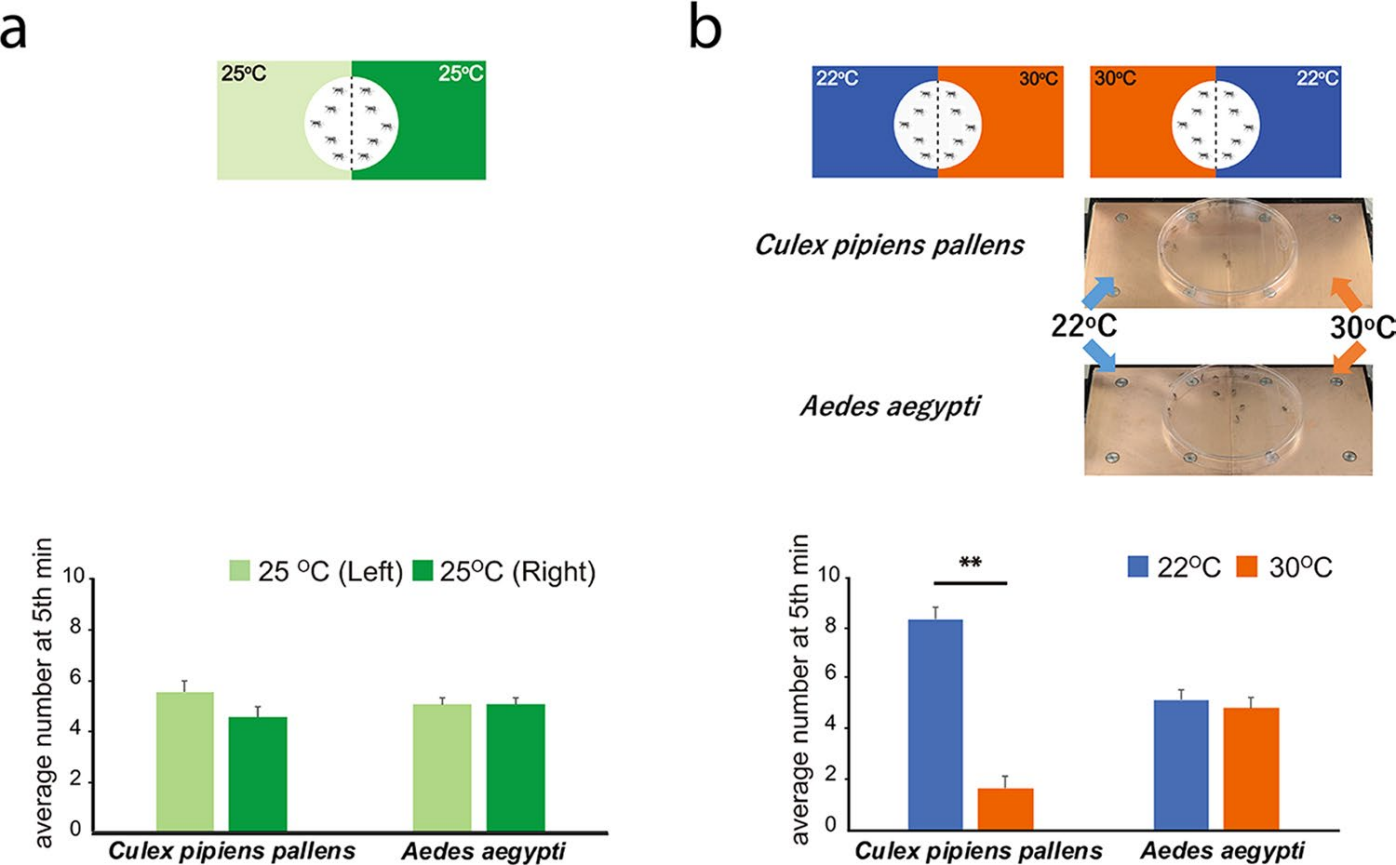
Comparison of temperature preference behaviors between *Culex pipiens pallens* and *Aedes aegypti*. (**a**) Schematic representation of the comparison between left and right side of the Peltier plate (25 °C). The average numbers of mosquito count at the 5^th^ minute in different areas of the Peltier plate are shown. n = 10 for each trial and 5 trials were performed. (**b**) Schematic representation of the comparison between 22 °C and 30 °C with representative results. The average numbers of mosquito count at the 5^th^ minute in different areas of the Peltier plate are shown. n = 10 for each trial and 5 trials were performed, **P < 0.01, unpaired Student’s *t*-test.

TRPA1 was reported to be crucial in tuning the thermotaxis in mosquitoes^7^. The aforementioned *in vitro* results imply that there might also be differences between mosquitoes inhabiting tropical and temperate regions in behavioral levels. To address this issue, we compared the behavioral difference between two mosquito species, *Culex pipiens pallens* and *Aedes aegypti*, since the greatest difference of temperature threshold between their TRPA1A channels (21.8 °C VS 32 °C) was found (Fig. 3b,c). Mosquitoes were anesthetized and restricted in a plastic dish with two equal zones with distinct temperature plates, 22 °C and 30 °C. They were allowed to move freely within this area and their distribution was counted every minute for five minutes. Intriguingly, significant difference of thermo-preference between *Culex pipiens pallens* and *Aedes aegypti* was observed after confirming no difference in the same temperature of 25 °C (Fig. 4a). The majority of *Culex pipiens pallens* gathered in the 22 °C zone while *Aedes aegypti* evenly distributed in the testing area, indicating they did not discriminate the difference between 22 °C and 30 °C (Fig. 4b). This behavioral difference between species suggests that the temperature thresholds for heat-evoked activation of TRPA1 of mosquitoes could be indicative of their respective thermotaxis *in vivo*.

### Splicing variants of mosquito TRPA1 also showed different chemical responses

**Figure 5:**
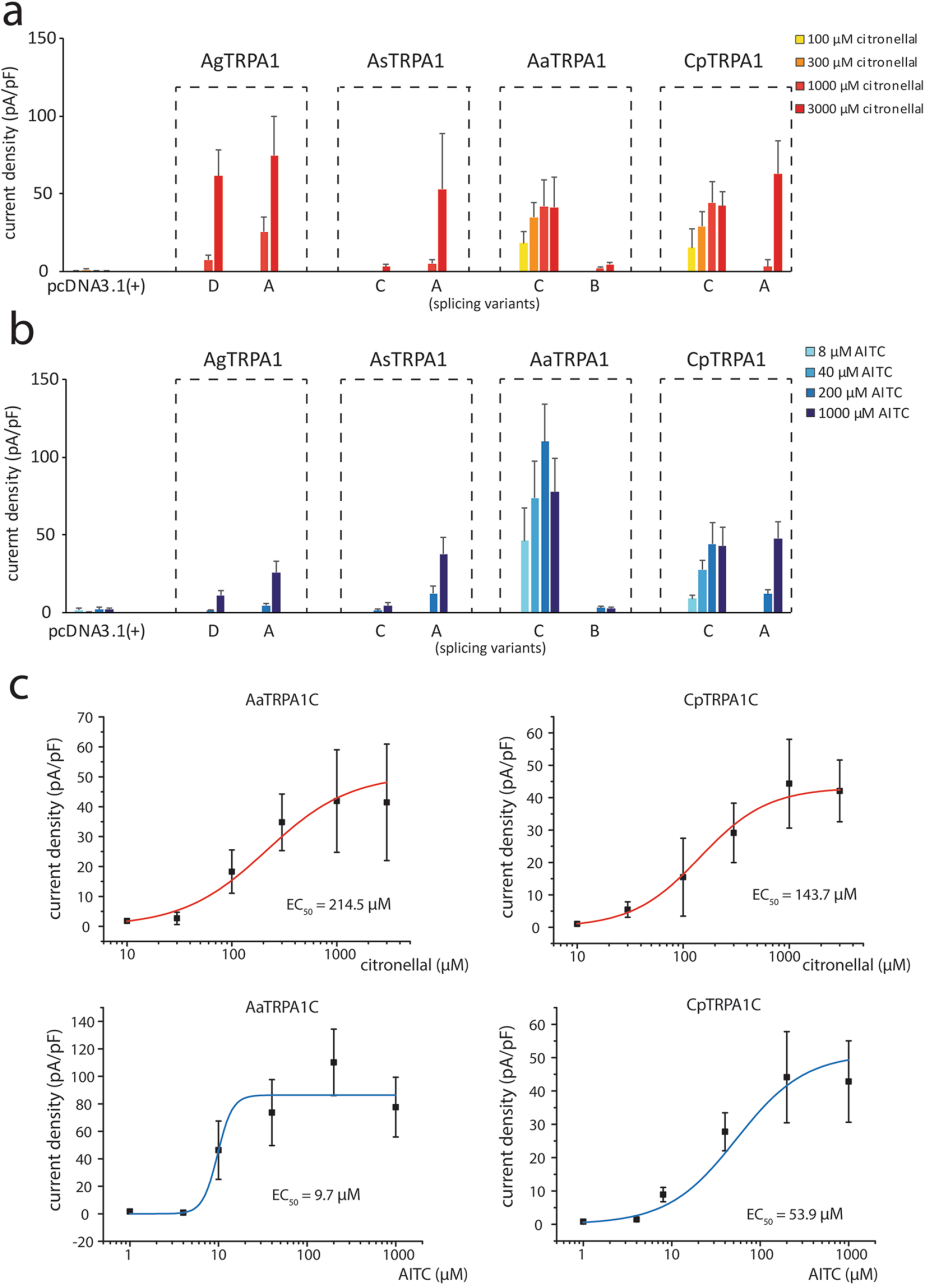
Comparison of chemosensitivities among TRPA1 variants and orthologues of mosquitoes. (**a**) Density comparison of the currents activated by 0.1, 0.3, 1 or 3 mM citronellal. (**b**) Density comparison of the currents activated by 0.008, 0.04, 0.2 or 1 mM AITC. All values are means ± S.E.M, 5 ≤ n ≤ 12 each. Disease vector mosquito TRPA1 variants were cloned and their function was examined with the classical mosquito repellent citronellal (**a**) and the conventional TRPA1 agonist AITC. (**b**) Peak current densities were measured with a whole-cell patch-clamp method. (**c**) Dose-dependent curves fit with *Hill*’s equation showing EC_50_ values of AaTRPA1C and CpTRPA1C upon citronellal or AITC administration. For each dot in the plot, n ≥ 6.

In parallel with testing the thermosensitivities of mosquito TRPA1s, we also characterized their chemosensitivities. AITC and citronellal are known TRPA1 agonists that reportedly induce feeding deterrence in *Drosophila melanogaster* through activation of TRPA1^6,15^, hereby the effects of the two compounds at increasing concentrations (AITC; 0.008 to 1 mM, citronellal; 0.1 to 3 mM) on splicing variants of multiple mosquito TRPA1s were investigated (Fig. S1c). There was no apparent difference in the responses to two chemicals in the examined mosquito TRPA1s, being consistent with the fact that both chemicals are electrophiles. More detailed comparative analyses demonstrated that AgTRPA1D and AgTRPA1A showed similar sensitivities to both chemicals, whereas the chemical sensitivity of AsTRPA1C was lower than that of AsTRPA1A (Fig. 5a,b). On the other hand, TRPA1C of *Aedes aegypti* and *Culex pipiens pallens* exhibited higher sensitivities to both chemicals than AaTRPA1B and CpTRPA1A, which was further confirmed by the EC_50_ values of TRPA1C of *Aedes aegypti* and *Culex pipiens pallens* upon citronellal or AITC administration (214.5 μM for citronellal, 9.7 μM for AITC for AaTRPA1C, and 143.7 μM for citronellal, 53.9 μM for AITC for CpTRPA1C) (Fig. 5c). When the sensitivities of both splicing variants of TRPA1 were combinatorically compared among species, TRPA1 of *Aedes aegypti* and *Culex pipiens pallens* had higher sensitivities compared to those of *Anopheles gambiae* and *Anopheles stephensi* (Fig. 5a,b). The difference of TRPA1 sensitivity to chemical stimuli is consistent with phylogeny of these four species that former and latter two species are closely related with each other as shown in Fig. S4a^21,22^.

### Expression levels and plasma membrane localization of AgTRPA1 in HEK293 cells

Splicing variants of TRPA1 showed variable responses to chemical and heat stimuli. These differences could result from both the variations in the expression levels on the cell membrane and/or channel properties of splicing variants of TRPA1. To quantify the amounts of TRPA1 proteins, Western blotting analyses were performed. Because it was not feasible to examine the expression levels in HEK293 cells of all the identified splicing variants of mosquito TRPA1, three representative TRPA1 variants of *Anopheles gambiae* (AgTRPA1D, AgTRPA1A(L) and AgTRPA1A(S)) were chosen for the comparison. Two Myc tags were added to the N-terminus of each TRPA1 channel protein with a double gly-gly-gly-ser linker in order to avoid interference of channel activity by addition of Myc tags (Fig. S1c).

The amounts of protein in the extracts from whole cells, cytoplasm and membranes of AgTRPA1D, AgTRPA1A(L) and AgTRPA1A(S) did not significantly differ (Fig. S5a,b). Moreover, responses of the AgTRPA1 channels to heat or citronellal were not affected by the addition of Myc tags, except for the heat-evoked currents of Myc-tagged AgTRPA1A(L) in which heat-evoked currents for Myc-tagged AgTRPA1A(L) were significantly larger than those for the untagged one (Fig. S5c).

To further investigate the plasma membrane expression of different variants, biotinylation assays were performed. Both AgTRPA1D and AgTRPA1A(L) were confirmed to translocate to the plasma membrane to a similar degree (Fig. S5d,e), which is consistent with the results in the Western blotting experiments (Fig. S5c,d). However, AgTRPA1A(S) showed higher membrane expression than did AgTRPA1A(L) (Fig. S5d,e), which was probably due to high expression in cells revealed by the higher expression in cell lysates. These results were again consistent with the regular Western blotting analysis (Fig. S5a,b), indicating the reliability of this result. The difference in the protein levels between AgTRPA1A(S) and AgTRPA1A(L) could explain the difference in the current densities between the two variants to some extent (Fig. 2).

### Screening of potential mosquito repellents

TRPA1 is well-known to be activated by electrophilic compounds such as AITC and cinnamaldehyde. However, these conventional TRPA1 agonists are pungent compounds that irritate the skin^23,24^, making them unappealing to most users. By quantifying the potencies of conventional TRPA1 agonists with a whole-cell patch-clamp method, mosquito TRPA1 currents evoked by citronellal were found comparable to those evoked by a strong electrophile, AITC (Fig. 5). Therefore, we screened many plant-derived compounds that are known to have anti-insect or anti-fungi efficacy to determine their effects on the activation of mosquito TRPA1 (Figs. S1c and S6).

**Figure 6:**
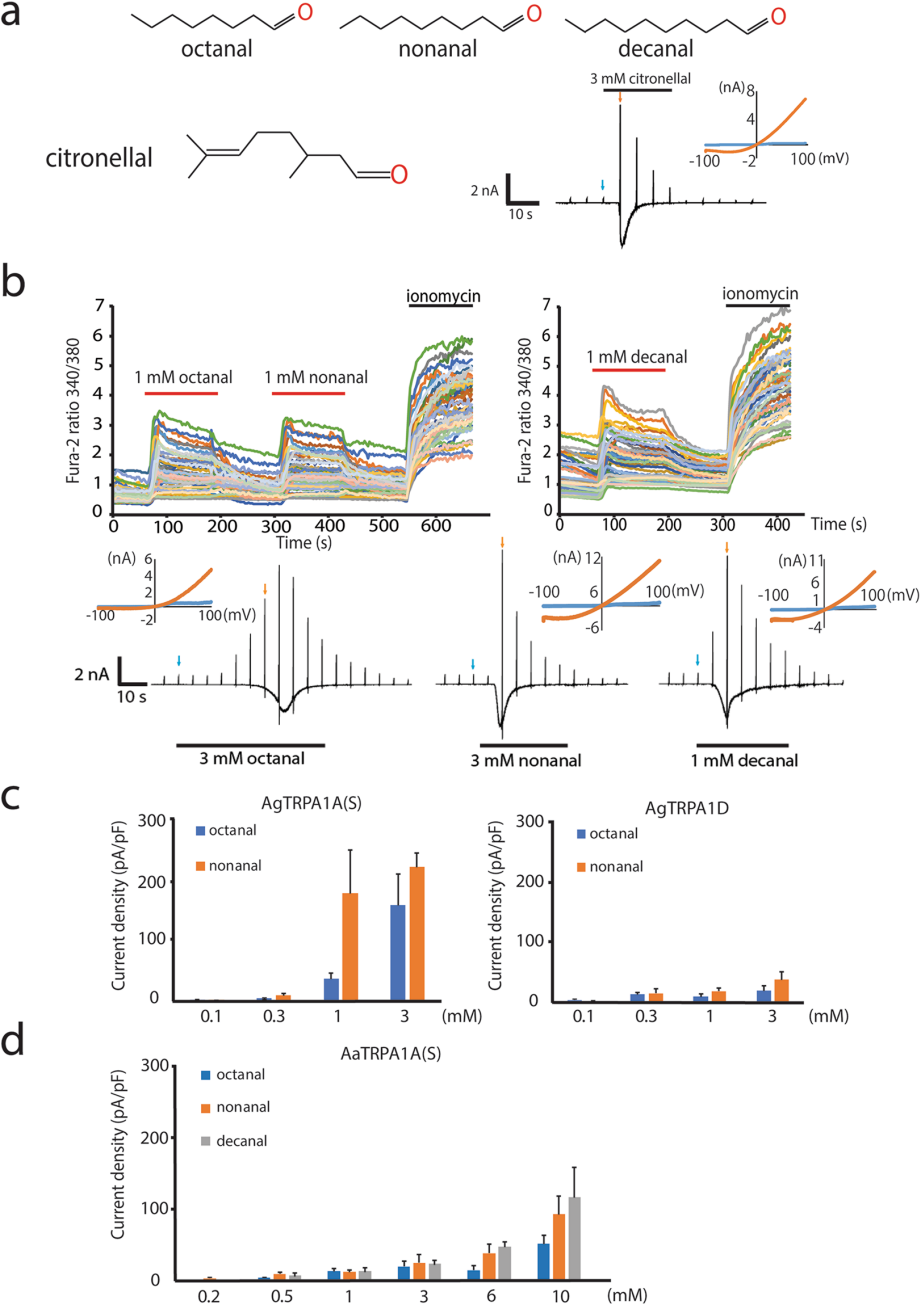
Molecular structures and effects of octanal, nonanal and decanal on mosquito TRPA1. (**a**) Molecular formulas of octanal, nonanal, decanal and citronellal. A representative trace of activation of AgTRPA1A(S) with 3 mM citronellal. (**b**) Octanal, nonanal and decanal were screened for their capacities to activate AgTRPA1A(S) with both a Fura-2 calcium-imaging method (upper) and a whole-cell patch-clamp method (lower). I–V curves are shown in insets. (**c**,**d**) Dose-dependent activation of AgTRPA1 by octanal and nonanal (**c**) and of AaTRPA1A(S) by octanal, nonanal and decanal (**d**). All values are means ± S.E.M; 4 ≤ n ≤ 10 each.

Twenty-two natural compounds were screened using a Fura-2 calcium-imaging technique. Octanal, nonanal and decanal were observed to robustly activate AgTRPA1 (Fig. S6). These compounds have been used commercially as components of perfumes and in flavoring agents for the food industry. Intriguingly, these three linear chain aldehydes have molecular structures similar to that of citronellal, and the difference is in the number of carbons in the main chain (Fig. 6a). AgTRPA1A(S) was chosen for this analysis since large currents were activated by citronellal through AgTRPA1A(S) (Fig. S1c). Outwardly rectifying AgTRPA1A(S)-mediated currents were rapidly activated by octanal, nonanal or decanal and inactivated even in the presence of these compounds as observed in the citronellal-evoked currents (Fig. 6a,b) possibly due to the desensitization after large activation.

The quantified whole-cell patch-clamp recordings indicated that these linear chain aldehydes activated AgTRPA1 in a dose-dependent manner, especially AgTRPA1A(S). Dose-dependent activation of AaTRPA1A(S) by the chemicals was also observed although the current densities were not as great as the ones seen with AgTRPA1A(S) (Figs. 6c,d and S1c), suggesting these compounds could act differently on the mosquito species.

## Discussion

Activation of TRPA1 causes pain sensation in mammals and avoidance behaviors in insects. Mosquitoes are the crucial vectors for transmission of malaria and other epidemic diseases in tropical and subtropical areas. *Anopheles*, *Aedes* and *Culex* are three representative genera of disease vector mosquitoes that are distributed across different regions of the world. TRPA1 was reported to be required for avoidance of high noxious temperatures in mosquitoes^7,8^. Moreover, mosquito TRPA1 is robustly activated by electrophiles and potentially serve as a target molecule for the development of effective repellents^10,15,20^. Physiological characteristics of TRPA1 are potentially changed during the speciation processes of mosquito lineages. Therefore, TRPA1 channel properties were systematically compared among four representative mosquito species.

In the present study, we identified several alternative splicing variants of the mosquito TRPA1 that possess distinct N-termini or different linker regions between the ankyrin repeats and the transmembrane domains (Fig. 1a). We also examined the genomic region of the same or closely related mosquito species in the *TrpA1* locus to identify which exons encoded these transcripts. Several highly conserved putative exons from *Anopheles stephensi*, *Aedes aegypti* and *Culex pipiens pallens* were determined. Unlike *AgTrpA1* in which alternative splicing of the N-terminal exons led to two different transcripts, two additional alternatively spliced downstream exons were discovered in the remaining three mosquito species. Besides, we discovered 14 amino acid at the N-terminus of mosquito TRPA1A which have not been recognized by the previous investigations (Fig. 1b).

Many regions of TRPA1 contribute to its sensitivity to thermal or chemical stimuli^25–28^. It was reported that the linker region is important for thermosensation in *Drosophila melanogaster* TRPA1^18^. While this comparison was conducted in *Aedes aegypti*, we did not observe a significant difference in heat-evoked responses (Fig. S3), suggesting that the linker region is not critical for temperature sensitivity in mosquitoes. Therefore, the observed differences in channel properties are probably due to the varying length of the N-termini as reported in insects^10,15,20^. This concept partly explains the significantly different channel activities observed in TRPA1A(L) and TRPA1A(S) in the N-terminal domain of mosquito TRPA1 (Figs. 1b and S1a).

Functional diversity exists in thermosensitive TRP channels. TRPA1 orthologues in rodents were first reported to be involved in cold-evoked events^2^, although their temperature sensitivity is currently controversial. However, many poikilotherm TRPA1s were reported to be activated by warm temperatures^14,29^. Even though these four mosquito species seem closely related, we readily observed differences in TRPA1 properties. First of all, TRPA1A (or TRPA1B) showed greater heat responses than did TRPA1C (or TRPA1D). This phenomenon mainly manifests in density of heat-evoked current (Fig. 3a), and it could be partly due to different tissue expression of TRPA1A (or TRPA1B) and TRPA1C (or TRPA1D) as in *Drosophila*^10^. Furthermore, TRPA1s of tropical *Anopheles* and *Aedes* mosquitoes had higher temperature thresholds, possessing a stronger thermal tolerance, than did *Culex pipiens pallens* that inhabits Northeast Asia (Fig. S4b)^30^. Indeed, *Culex pipiens pallens* likely sense the noxious ambient temperature over 30 °C and avoided it. In contrast, *Aedes aegypti* did not distinguish the difference between 22 °C and 30 °C (Fig. 4). Such a weaker temperature preference might be consistent in other tropical mosquitoes investigated in this study. Taken together, these findings indicate that the functional changes of thermosensation in TRPA1s potentially depend on the adaptation to different thermal niches although it is not clear that TRPA1 is a sole arbiter of the heat-responsive behaviors in the mosquitoes.

Arthropod species have the most diversified TRPA1 subfamily members because they exhibit extensive evolutionary plasticity^31^. The evolutionary relationship of the disease vector mosquitoes investigated in this study were previously analyzed^22^. *Anopheles gambiae* and *Anopheles stephensi* are closely related sister taxa that split about 30 mya. On the other hand, *Aedes aegypti* and *Culex pipiens* diversified about 57 mya (Fig. S4a)^21^. Two major differences could be assumed between these mosquito species from 2 clades. First, there might be differences between these 2 clades in their abilities to sense chemicals. AaTRPA1 and CpTRPA1 were capable of detecting very low concentrations of AITC and citronellal whereas *Anopheles* TRPA1 could not (Fig. 5). Second, AaTRPA1C or CpTRPA1C were found to be more chemosensitive than AaTRPA1B or CpTRPA1A, respectively, while such clear difference was not found in *Anopheles* mosquitoes. Likewise, AaTRPA1 and CpTRPA1 had higher chemosensitivities compared to those of *Anopheles* mosquitoes. However, the amino acid alignment of TRPA1 indicates that the previously reported critical conservative cysteine residues located in the ankyrin repeat domain are mostly conserved in all the variants of the four mosquito species (Fig. S1b). Therefore, there might be other crucial residues that play important roles in modulating and regulating chemical sensitivity in TRPA1. In addition, difference in the pattern of thermal and chemical sensitivity among four mosquito species can be partly explained by their phylogenetic relationship. Amino acid changes that increased chemical sensitivity of TRPA1 may took place in the common ancestor of *Aedes aegypti* and *Culex pipiens* after the split from *Anopheles* mosquitos, which did not affect thermal sensitivity of TRPA1. In other words, identification of such amino acid responsible for the observed species difference might provide us with a clue for understanding the structural basis of TRPA1 action mechanisms in the future.

Diverse current sizes could be the consequence of different expression levels of TRPA1 variants. We measured the expression levels of Myc-tagged AgTRPA1s and compared their channel properties with untagged ones (Fig. S5). The negligible thermosensitivity of TRPA1D was not explained by its low expression on the cell membrane. The possibility that differences between longer and shorter TRPA1A results from different expression levels cannot be ruled out by comparing the protein expression of AgTRPA1A(L) and AgTRPA1A(S) because the difference in current densities was parallel to the membrane expression levels (Figs. 2 and S5e) although the temperature thresholds were not different between longer and shorter variants both in AgTRPA1 and AaTRPA1B (Fig. 3d). The biotinylating assay revealed distinct expression levels of AgTRPA1A(L) and AgTRPA1A(S) on the HEK293 cell membrane which could explain why both thermosensitivity and chemosensitivity of AgTRPA1A(L) were significantly lower. However, the mechanism by which the 14 amino acids at the N-terminus of TRPA1 alter the expression level of TRPA1 on the plasma membrane remains to be clarified. Taken together, these results highlighted the importance of the TRPA1 N-terminus for thermal sensitivity.

This investigation identified several TRPA1 alternative splice variants from four disease vector mosquito species. The variants were characterized in detail by combinatorically comparisons. A structural determinant for modulating channel properties was revealed, and the thermosensitivity of TRPA1 could reflect the thermal tolerance for the climate of specific ecological niches. These results could be extended using mosquitoes for behavioral analysis to unravel the mechanism by which TRPA1 functions in the regulation of mosquito host-seeking and avoidance behaviors.

## Methods

### Animals

Adult mosquito samples of wild-type *Anopheles stephensi*, wild-type *Aedes aegypti*, wild-type *Culex pipiens pallens* and pupa of wild-type *Aedes aegypti* for molecular cloning were reared in laboratory (Jikei University School of Medicine, Tokyo, Japan). Female adult *Culex pipiens pallens* 3 to 4 days after eclosion and female adult *Aedes aegypti* 5 to 6 days after eclosion were used the behavioral tests. *Culex pipiens pallens* was kept at 26 °C temperature and 50% of relative humidity under 12 hrs light/12 hrs dark condition fed with 3% yellow soft sugar. *Aedes aegypti* was kept at 28 °C temperature and 60% of relative humidity under 12 hrs light/12 hrs dark condition fed with 3% yellow soft sugar.

### 5′- and 3′- RACE of mosquito *TrpA1*

5′- and 3′-RACE was performed with SMARTer RACE 5′/3′Kit (Takara, Kyoto, Japan) to identify the splicing variants of TRPA1 from three mosquito species. Gene specific primers used for 5′- and 3′-RACE were as shown in Supplementary Table S1a.

### Construction of mosquito TRPA1-expressing vector for mammalian cells

*TrpA1* variants were cloned into the pcDNA3.1 vector. Primers were designated for each *TrpA1* splicing variants to perform nested PCR to obtain DNA fragments containing entire coding region of TRPA1 (Supplementary Table S1b). For Myc-tagged vector construction, primers were designated for amplifying 2Myc-C-*AgTrpA1*-pcDNA3.1(+) (Supplementary Table S1c), which were further used as templates for amplifying linear Myc-tagged *AgTrpA1* with double gly-gly-gly-ser linkers, the respective primers were as shown in Supplementary Table S1c.

### Cell culture

The human embryonic kidney 293 (HEK293) cells were cultured in Dulbecco’s modified Eagle Medium (Wako, Tokyo, Japan) supplemented with 10% fetal bovine serum (Gibco, Grand Island, NY, USA), penicillin-streptomycin (50 mg/mL and 50 units/mL, respectively, Gibco) and GlutaMAX (2 mM, Gibco). For transient transfection of HEK293 cells, 1 μg of plasmid DNA in pcDNA3.1 (+) and 0.1 μg pGreen-Lantern 1 vector were transfected into HEK293 cells using Lipofectamine reagent and Plus reagent (Invitrogen, Carlsbad, CA, USA). In the case of transfection for calcium-imaging, 0.1 μg pCMV-DsRed vector was transfected instead of pGreen-Lantern 1 vector. All these components were dissolved in OPTI-MEM medium (1X, Gibco). After incubation for 3–4 h, HEK293 cells were reseeded on 12-mm cover slips (Matsunami, Tokyo, Japan) and further incubated at 33 °C in 5% CO_2_.

### Calcium imaging and electrophysiology

Both calcium-imaging and whole-cell patch-clamp recording experiments were performed 18–30 h after transfection. The extracellular standard bath solution contained 140 mM NaCl, 5 mM KCl, 2 mM MgCl_2_, 2 mM CaCl_2_, 10 mM HEPES and 10 mM glucose at pH 7.4, adjusted with NaOH. Cytosolic-free Ca^2+^ concentrations were measured with Fura-2 (Molecular Probes, Invitrogen Corp). Fura-2-AM (5 μM) was loaded 1 h before recording, and it was excited at 340/380 nm with emission at 510 nm. Fura-2 fluorescence was recorded with a CCD camera, Cool Snap ES (Roper Scientific/Photometrics, Vianen, Bilthoven, Netherlands). Data were acquired using imaging processing software (IPlab Scanalytics, Milwaukede, WI, USA) and analyzed with ImageJ (RRID:SCR_003070; National Institutes of Health, Bethesda, MD, USA). For whole-cell patch-clamp recording, the intracellular pipette solution contained 140 mM KCl, 5 mM EGTA, and 10 mM HEPES at pH 7.4 adjusted with KOH. For assessment of peptide overexpression, the stock solution (10 mM) was added into the pipette solution to prepare different concentrations. Recording started 2 to 3 min after making a whole-cell configuration to achieve steady state^32^. The data from whole-cell patch-clamp recordings were acquired at 10 kHz and filtered at 5 kHz for analysis (Axopatch 200B amplifier with pCLAMP software, Molecular Devices, Sunnyvale, CA, USA). Membrane potential was clamped at −60 mV and voltage ramp-pulses from −100 to +100 mV (0.5 s) were applied every 5 s. For heat-stimulation, the cell was gently lifted up and placed in the center of the chamber after forming a whole-cell configuration. The ambient temperature of the patch-clamped HEK293 cell was measured with a thermocouple (TA-30; Warner Instruments). Current density (pA/pF) was calculated as the quotient of current amplitude (pA) divided by whole cell capacitance (pF). Arrhenius plots were generated with Origin 8.1 (Microcal, Northampton, MA, USA). The temperature coefficient Q_10_ was for characterizing the temperature dependent membrane current. The absolute current values were plotted on a log scale against the reciprocal of the absolute temperature (*T*) (from Arrhenius plot). Q_10_ values were calculated from Q_Δ*T*_ = (Q_10_)^Δ*T*/10^ for an arbitrary temperature Δ*T*^33^.

### Temperature choice test

Tested mosquitoes are *Culex pipiens pallens* (female adult) and *Aedes aegypti* (female adult). Experiments were performed under the stable conditions of ambient temperature (25.0–26.2 °C) and humidity (33–38%). 10 tested insects were cryanesthetized onto a piece of weighing paper and they were covered with a plastic dish (90 mm diameter and 9 mm depth). The anesthetized mosquitoes were transferred to the center of the Peltier plate. Afterwards, the awaken mosquitoes were allowed to make the choice between left or right side of the Peltier plate with different temperatures. The observation lasted for 5 minutes, and the number of mosquitoes on left and right sides was counted every minute. The temperature setting on the left and right sides of the Peltier plate was switched for different batches of observation (Fig. 4).

### Protein isolation

Whole cell extracts of HEK293T cells transfected with 4 μg of plasmid DNA were collected by scraping in RIPA lysis buffer (Cell Signaling Technology, Danvers, MA, USA). Cytosolic and membrane fractions were isolated using a subcellular protein fractionation kit (Thermo Fisher Scientific, Waltham, MA, USA). Protein concentration was quantified with the Lowry assay (Bio-Rad, Hercules, CA, USA). Proteins (30 μg) from whole cell, cytosolic and membrane fractions were denatured with 4x Laemmli Sample Buffer (Bio-Rad) and 100 mM DTT at 95 °C for 5 min before resolution using an 8% SDS-PAGE gel.

### Plasma membrane protein biotinylation

The HEK293 cells transfected with 1 μg of plasmid DNA harboring Myc-tagged TRPA1 or empty plasmid DNA were incubated twice with 0.5 mg/mL EZ-link-NHS-LC-Biotin (Abcam, Cambridge, UK). Biotinylated proteins were precipitated overnight using 10 μL Dynabeads MyOne Streptavidin T1 (Thermo Fisher Scientific) with agitation at 4 °C.

### Western blotting

Primary antibodies were used to incubate the membrane overnight. They included: anti-Myc (MBL Life science, Nagoya, Aichi, Japan): 1/5000; anti-β-actin (Sigma-Aldrich, St. Louis, MO, USA): 1/2000 diluted in PBS-T containing 1.5% BSA for isolated protein samples; anti-Myc: 1/1000 diluted in PBS-T containing 1.5% BSA for biotinylated protein samples). Protein bands were detected with the use of Amersham ECL Prime Western Blotting Detection Reagent (GE Healthcare, Waukesha, WI, USA) and a LAS-3000 mini image analyzer (Fujifilm, Tokyo, Japan). Quantification of Western blot bands was performed using Image J software.

### Statistical analysis

Data are presented as means ± standard error of mean (S.E.M). Statistical analysis was performed by unpaired Student’s *t*-test or one-way ANOVA with Bonferroni’s *post hoc* analysis. P < 0.05 was considered to be significant. Statistical significance is defined as: *p < 0.05; **p < 0.01 and ***p < 0.001.

### Accession codes

The nucleotide sequences for the *AaTrpA1C*, *AaTrpA1A*, *AaTrpA1B*, *AsTrpA1C*, *AsTrpA1A*, *CpTrpA1C* and *CpTrpA1A* splice variants reported in this paper have been deposited in DDBJ under accession codes LC438794, LC438795, LC438796, LC438797, LC438798, LC438799 and LC438800, respectively.

## Supplementary information

Supplementary Information.
