# Supplementary material for: Diverse sensitivities of TRPA1 from different mosquito species to thermal and chemical stimuli
Source: Sci Rep. 2019 Dec 27;9:20200. doi: 10.1038/s41598-019-56639-w (PMC6934445; doi:10.1038/s41598-019-56639-w)
Supplement: Supplementary file 1 — Supplementary Information. [file 41598_2019_56639_MOESM1_ESM.pdf]

Title:

Diverse sensitivities of TRPA1 from different mosquito species to thermal and chemical stimuli

Author names and affiliations:

Tianbang Li[1,2,3], Claire T. Saito[2,3], Tomoyuki Hikitsuchi[4], Yoshihiro Inoguchi[4], Honami Mitsuishi[4], Shigeru Saito[1,2,3]*, Makoto Tominaga[1,2,3] *

*Correspondence: tominaga@nips.ac.jp, sshigeru@nips.ac.jp

[1]Department of Physiological Sciences, SOKENDAI, Okazaki, Japan

[2]Division of Cell Signaling, National Institute for Physiological Sciences, Okazaki, Japan

[3]Thermal Biology Group, Exploratory Research Center on Life and Living Systems (ExCELLS), Okazaki, Japan

[4]Research & Development Laboratory, Dainihon Jochugiku Co., Ltd., Osaka, Japan

Figure S1. DNA sequence alignment and amino acid sequence alignment of mosquito TRPA1s

(a) DNA sequence alignment showing the beginning of the *TrpA1A* gene from 4 mosquito species. Nucleotides are aligned in triplets, and the codons from the exon region are translated into amino acids listed below. Three critical methionine codons are highlighted red, and the upstream stop codons are highlighted green.

(b) Amino acid sequence alignment of TRPA1 splicing variants from 4 mosquito species. Ankyrin repeats are labeled with black and grey bars, a linker region with a red bar, transmembrane domains with blue bars, and a pore loop with a green bar. Asterisks note the reported conserved residues for electrophile detection.

(c) Cartoons indicate specific mosquito TRPA1 transcripts analyzed in different experiments.

Figure S2. Effects of the rate of temperature change on the heat-evoked activation of mosquito TRPA1

(a) An example of a linear 10_{o}C temperature rise during TRPA1 activation (left). The adopted temperature trace is highlighted with red color. An Arrhenius plot of the current evoked by heat stimulation is shown based on the left trace (right).

(b) There was a positive correlation between the rate of temperature increase and the heat-evoked current density (left), whereas there was no correlation between the rate of temperature increase and the temperature threshold for heat activation. All the data were recorded and measured from HEK293 cells expressing AgTRPA1A(S). R-squared values are shown in both scatter diagrams.

Figure S3. AaTRPA1A and AaTRPA1B possess similar thermosensitivities

(a) Changes in cytosolic calcium concentrations (upper) in response to temperature elevation

(lower) or AITC application in HEK293 cells expressing AaTRPA1A (left) or AaTRPA1B (right).

(b) Density comparison of the currents activated by increasing bath solution temperature from 15°C to near 42°C using HEK293 cells expressing AaTRPA1A or AaTRPA1B in whole-cell patch-clamp recording. All values are means ± S.E.M, n = 9 for AaTRPA1A, n = 21 for AaTRPA1B, unpaired Student's *t*-test.

Figure S4. Phylogenic tree and distribution map of the four mosquito species investigated

(a) A phylogenic tree and the estimated divergence dates of the four mosquito species investigated. Figure is a simplified version based on the publication of Kamali et al. Nodes are at mean divergence time (in millions of years with standard errors).

(b) Distribution map of the four mosquito species investigated. Different colors of the areas of distribution correspond to different mosquito species, red for *Anopheles gambiae*, brown for *Anopheles stephensi*, purple for *Aedes aegypti*, blue for *Culex pipiens pallens*. Blue lines indicate the habitats of *Culex* mosquitoes. The habitats of mosquitoes overlap with each other.

Figure S5. Expression levels and plasma membrane localization of variants of AgTRPA1 in HEK293 cells analyzed with Western blotting

(a) Full-length blots of representative protein expression of Myc-tagged AgTRPA1 in whole cell lysate (whole), cytoplasm and membrane. 1/5000 diluted anti-Myc and 1/2000 diluted anti-β-actin primary antibodies were used to incubate the upper blot and the lower blot, respectively.

(b) Quantified analysis of Myc-tagged AgTRPA1 in whole cell lysate (whole), cytoplasm and membrane. All values normalized to β-actin are means ± S.E.M, n = 3. No significant difference, one-way ANOVA with Bonferroni's *post hoc* analysis.

(c-e) Comparison of the 3 mM citronellal-evoked (left) and heat-evoked (right) currents in HEK293 cells expressing AgTRPA1D, AgTRPA1A(L) and AgTRPA1A(S) with or without Myc tags.

All values are means ± S.E.M; $5 \leq n \leq 20$ each; **, $P < 0.01$, unpaired Student's t-test. (d) Representative biotinylated AgTRPA1 membrane protein (pull) and the eluate of cell lysate (input), and comparison by the quantitative analysis (e).

Figure S6.

Representative calcium imaging traces for screening the effects of the 22 chemicals on AgTRPA1A(S).

Each trace represents one DsRed positive HEK239 cell, indicating the transfection was successful.

Table S1. Primer sequences used in this research

(a) Primer sequences designed for the 5' and 3' RACE of TrpA1 of *Anopheles stephensi, Aedes aegypti* and *Culex pipiens pallens*.

(b) Primer sequences designed for the amplification of TrpA1 of *Anopheles stephensi*, *Aedes aegypti* and *Culex pipiens pallens*. And primer sequences designed for subcloning *TrpA1(A)* and *TrpA1(A)* of *Anopheles gambiae*.

(c) Primer sequences designed for the amplification of two Myc-tagged *Anopheles gambiae TrpA1* variants with a double gly-gly-gly-ser linker.

a

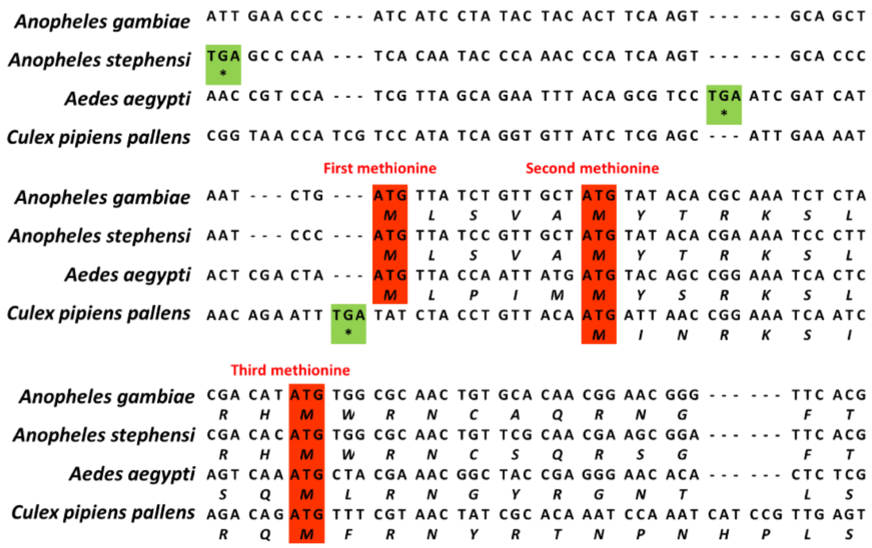
Anopheles gambiae ATT GAA CCC --- ATC ATC CTA TAC TAC ACT TCA AGT --- --- GCA GCT
Anopheles stephensi TGA GCC CAA --- TCA CAA TAC CCA AAC CCA TCA AGT --- --- GCA CCC
*
Aedes aegypti AAC CGT CCA --- TCG TTA GCA GAA TTT ACA GCG TCC TGA ATC GAT CAT
*
Culex pipiens pallens CGG TAA CCA TCG TCC ATA TCA GGT GTT ATC TCG AGC --- ATT GAA AAT
First methionine
Second methionine
Anopheles gambiae AAT --- CTG --- ATG TTA TCT GTT GCT ATG TAT ACA CGC AAA TCT CTA
M L S V A M Y T R K S L
Anopheles stephensi AAT --- CCC --- ATG TTA TCC GTT GCT ATG TAT ACA CGA AAA TCC CTT
M L S V A M Y T R K S L
Aedes aegypti ACT CGA CTA --- ATG TTA CCA ATT ATG ATG TAC AGC CGG AAA TCA CTC
M L P I M M Y S R K S L
Culex pipiens pallens AAC AGA ATT TGA TAT CTA CCT GTT ACA ATG ATT AAC CGG AAA TCA ATC
* M I N R K S I
Third methionine
Anopheles gambiae CGA CAT ATG TGG CGC AAC TGT GCA CAA CGG AAC GGG --- --- TTC ACG
R H M W R N C A Q R N G F T
Anopheles stephensi CGA CAC ATG TGG CGC AAC TGT TCG CAA CGA AGC GGA --- --- TTC ACG
R H M W R N C S Q R S G F T
Aedes aegypti AGT CAA ATG CTA CGA AAC GGC TAC CGA GGG AAC ACA --- --- CTC TCG
S Q M L R N G Y R G N T L S
Culex pipiens pallens AGA CAG ATG TTT CGT AAC TAT CGC ACA AAT CCA AAT CAT CCG TTG AGT
R Q M F R N Y R T N P N H P L S

Fig. S1

b

Fig. S1

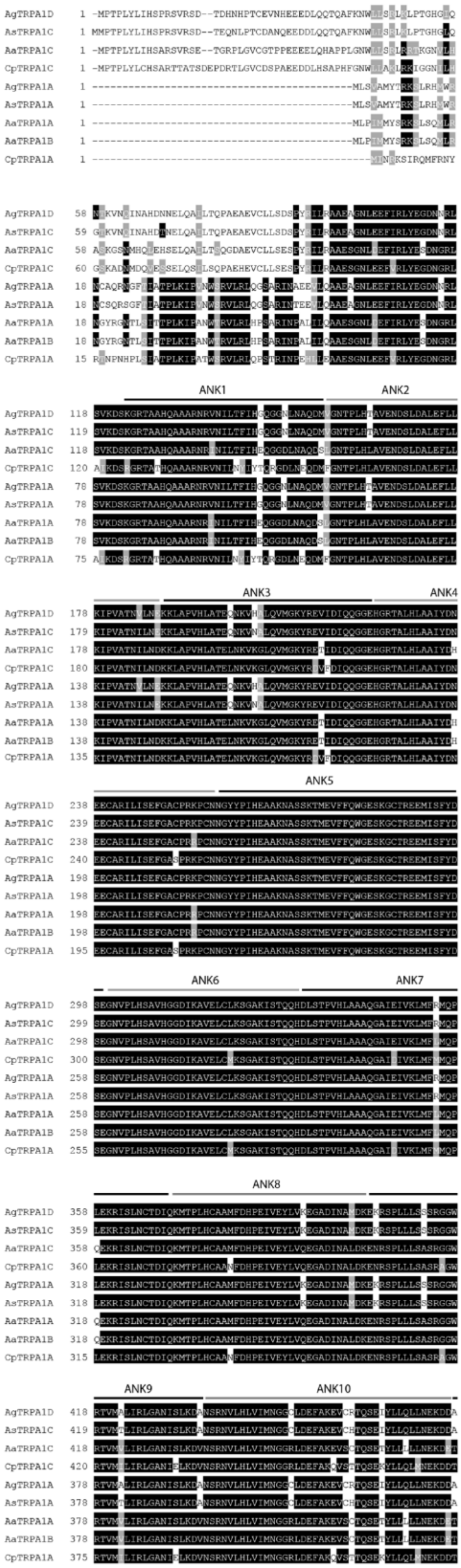
AgTRPA1D
AsTRPA1C
AaTRPA1C
CpTRPA1C
AgTRPA1A
AsTRPA1A
AaTRPA1A
AaTRPA1B
CpTRPA1A
ANK1
ANK2
ANK3
ANK4
ANK5
ANK6
ANK7
ANK8
ANK9
ANK10

Fig. S1

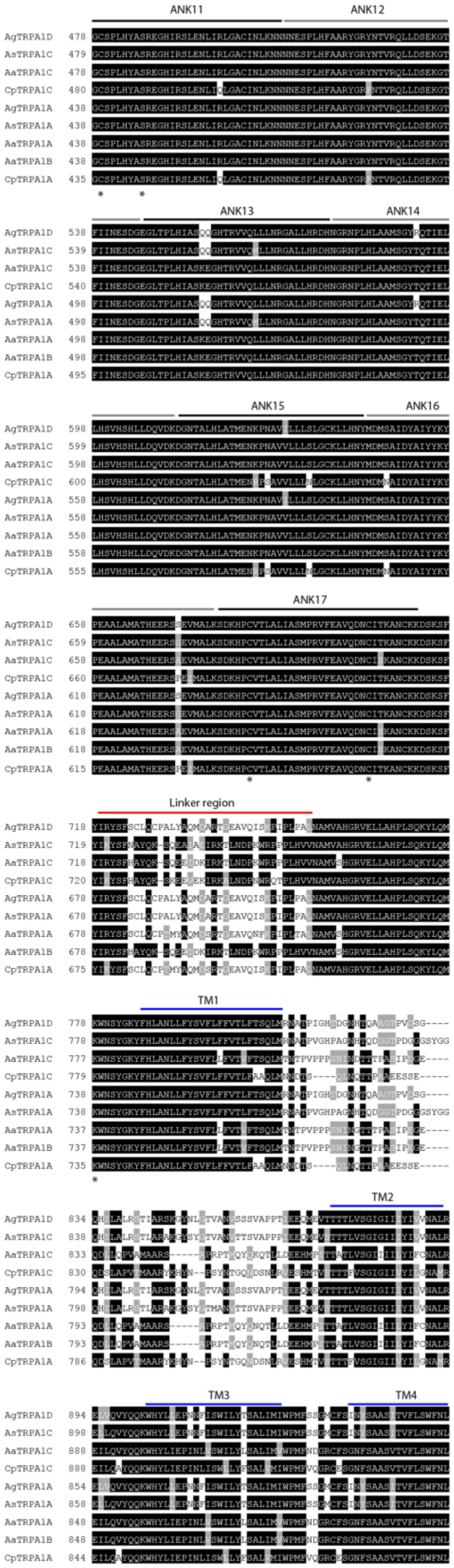
ANK11
ANK12
ANK13
ANK14
ANK15
ANK16
ANK17
Linker region
TM1
TM2
TM3
TM4

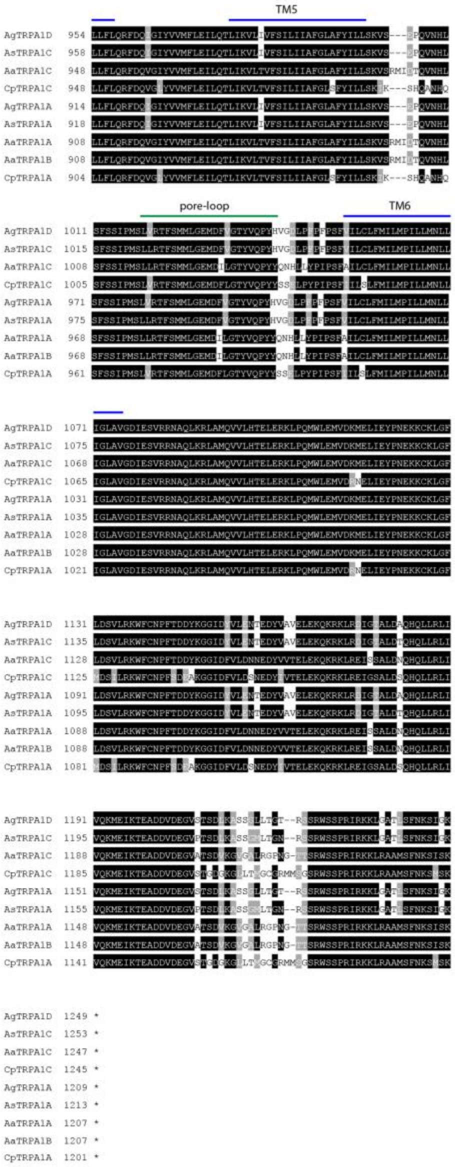

Fig. S1

c

Fig. S1

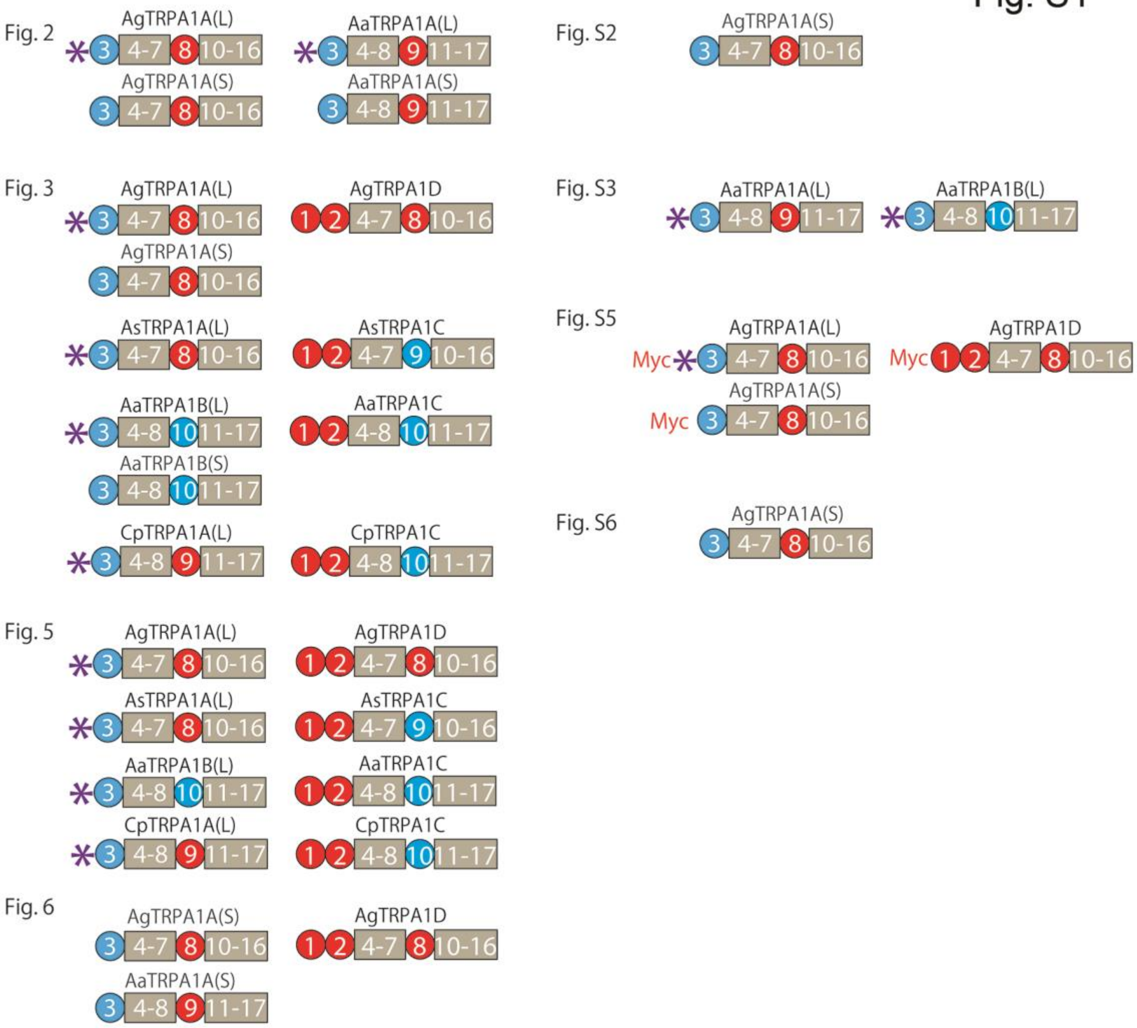

Fig. 2
AgTRPA1A(L)
3 4-7 8 10-16
AgTRPA1A(S)
3 4-7 8 10-16
AaTRPA1A(L)
3 4-8 9 11-17
AaTRPA1A(S)
3 4-8 9 11-17
Fig. 3
AgTRPA1A(L)
3 4-7 8 10-16
AgTRPA1A(S)
3 4-7 8 10-16
AgTRPA1D
1 2 4-7 8 10-16
AsTRPA1A(L)
3 4-7 8 10-16
AsTRPA1C
1 2 4-7 9 10-16
AaTRPA1B(L)
3 4-8 10 11-17
AaTRPA1B(S)
3 4-8 10 11-17
AaTRPA1C
1 2 4-8 10 11-17
CpTRPA1A(L)
3 4-8 9 11-17
CpTRPA1C
1 2 4-8 10 11-17
Fig. 5
AgTRPA1A(L)
3 4-7 8 10-16
AgTRPA1D
1 2 4-7 8 10-16
AsTRPA1A(L)
3 4-7 8 10-16
AsTRPA1C
1 2 4-7 9 10-16
AaTRPA1B(L)
3 4-8 10 11-17
AaTRPA1C
1 2 4-8 10 11-17
CpTRPA1A(L)
3 4-8 9 11-17
CpTRPA1C
1 2 4-8 10 11-17
Fig. 6
AgTRPA1A(S)
3 4-7 8 10-16
AaTRPA1A(S)
3 4-8 9 11-17
AgTRPA1D
1 2 4-7 8 10-16
Fig. S2
AgTRPA1A(S)
3 4-7 8 10-16
Fig. S3
AaTRPA1A(L)
3 4-8 9 11-17
AaTRPA1B(L)
3 4-8 10 11-17
Fig. S5
AgTRPA1A(L)
Myc 3 4-7 8 10-16
AgTRPA1A(S)
Myc 3 4-7 8 10-16
AgTRPA1D
Myc 1 2 4-7 8 10-16
Fig. S6
AgTRPA1A(S)
3 4-7 8 10-16

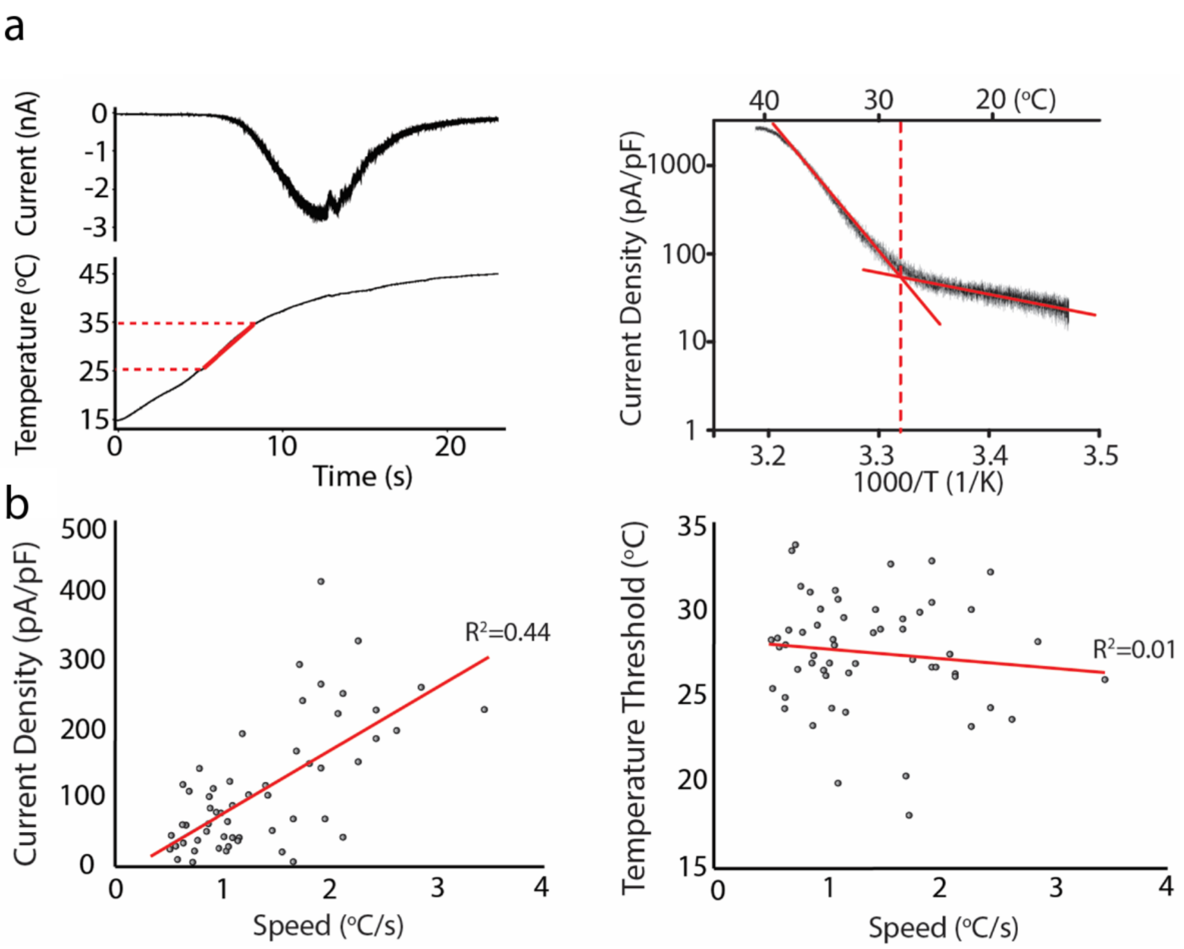
a
Current (nA)
0
-1
-2
-3
Temperature (°C)
45
35
25
15
0
10
20
Time (s)
40
30
20 (°C)
Current Density (pA/pF)
1000
100
10
1
3.2
3.3
3.4
3.5
1000/T (1/K)
b
Current Density (pA/pF)
500
400
300
200
100
0
R²=0.44
0
1
2
3
4
Speed (°C/s)
Temperature Threshold (°C)
35
30
25
20
15
R²=0.01
0
1
2
3
4
Speed (°C/s)

Fig. S2

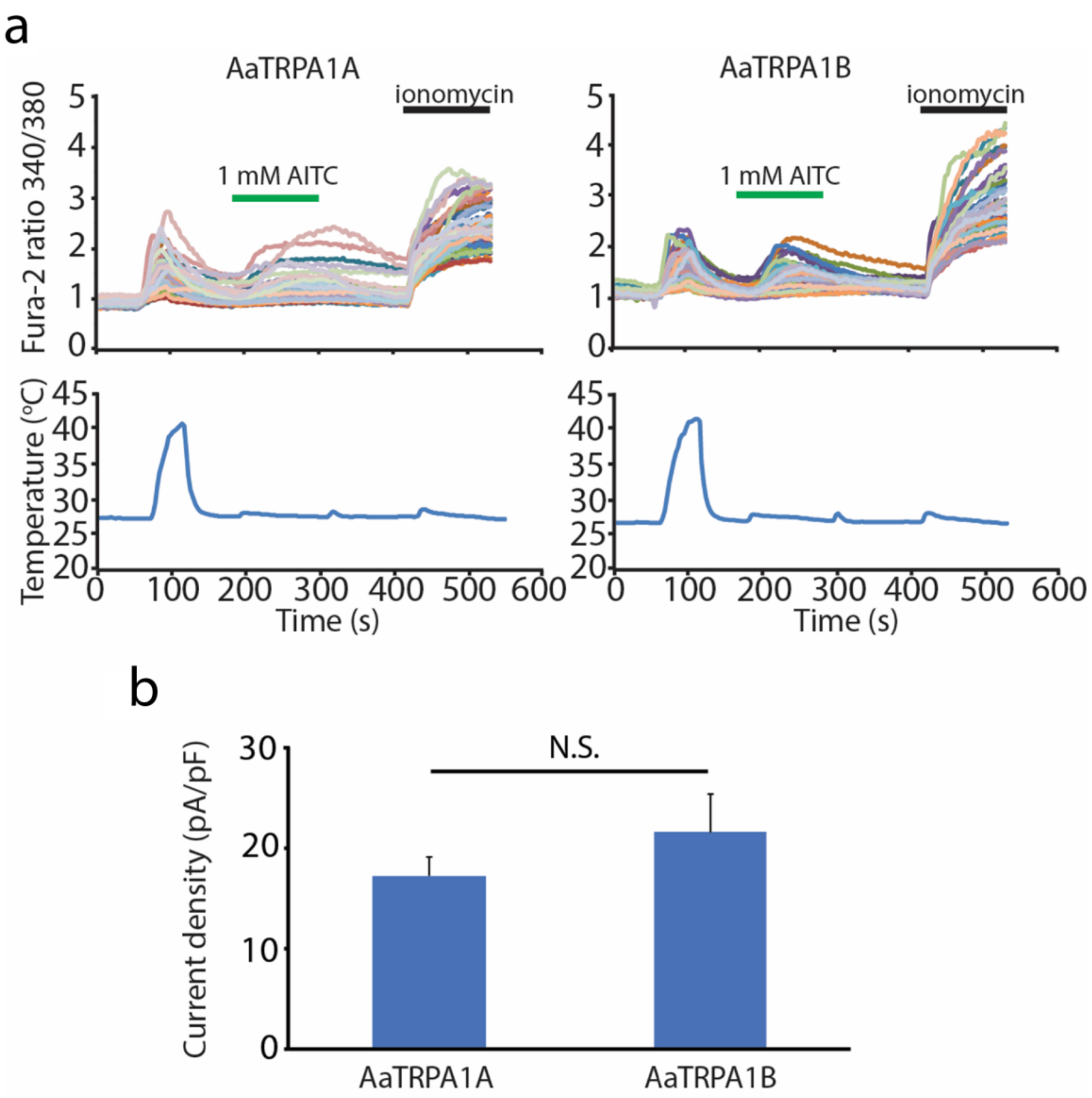
a
AaTRPA1A
AaTRPA1B
ionomycin
1 mM AITC
Fura-2 ratio 340/380
Temperature (°C)
Time (s)
b
N.S.
Current density (pA/pF)
AaTRPA1A
AaTRPA1B

Fig. S3

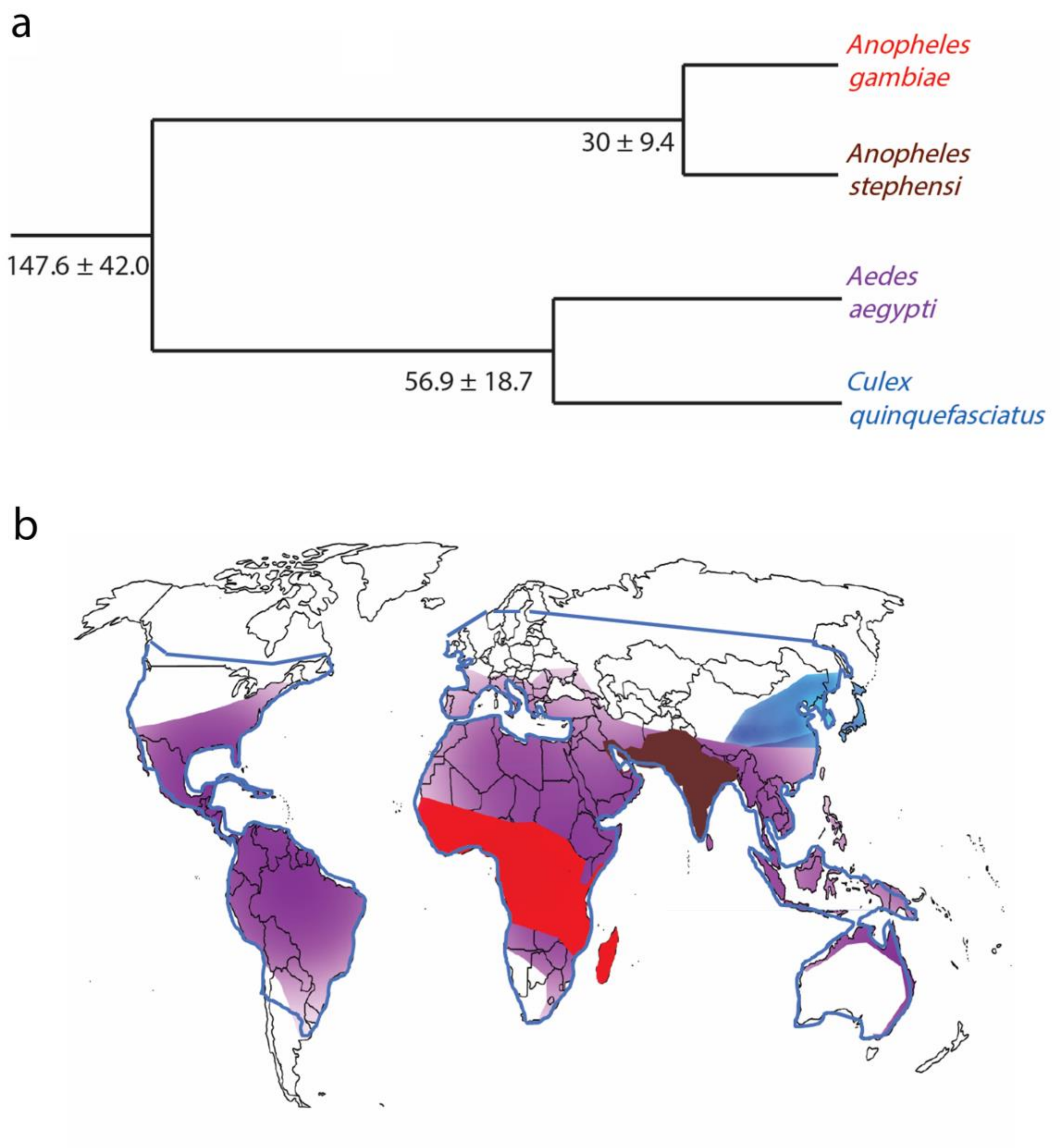
a
Anopheles gambiae
30 ± 9.4
Anopheles stephensi
147.6 ± 42.0
Aedes aegypti
56.9 ± 18.7
Culex quinquefasciatus
b

Fig. S4

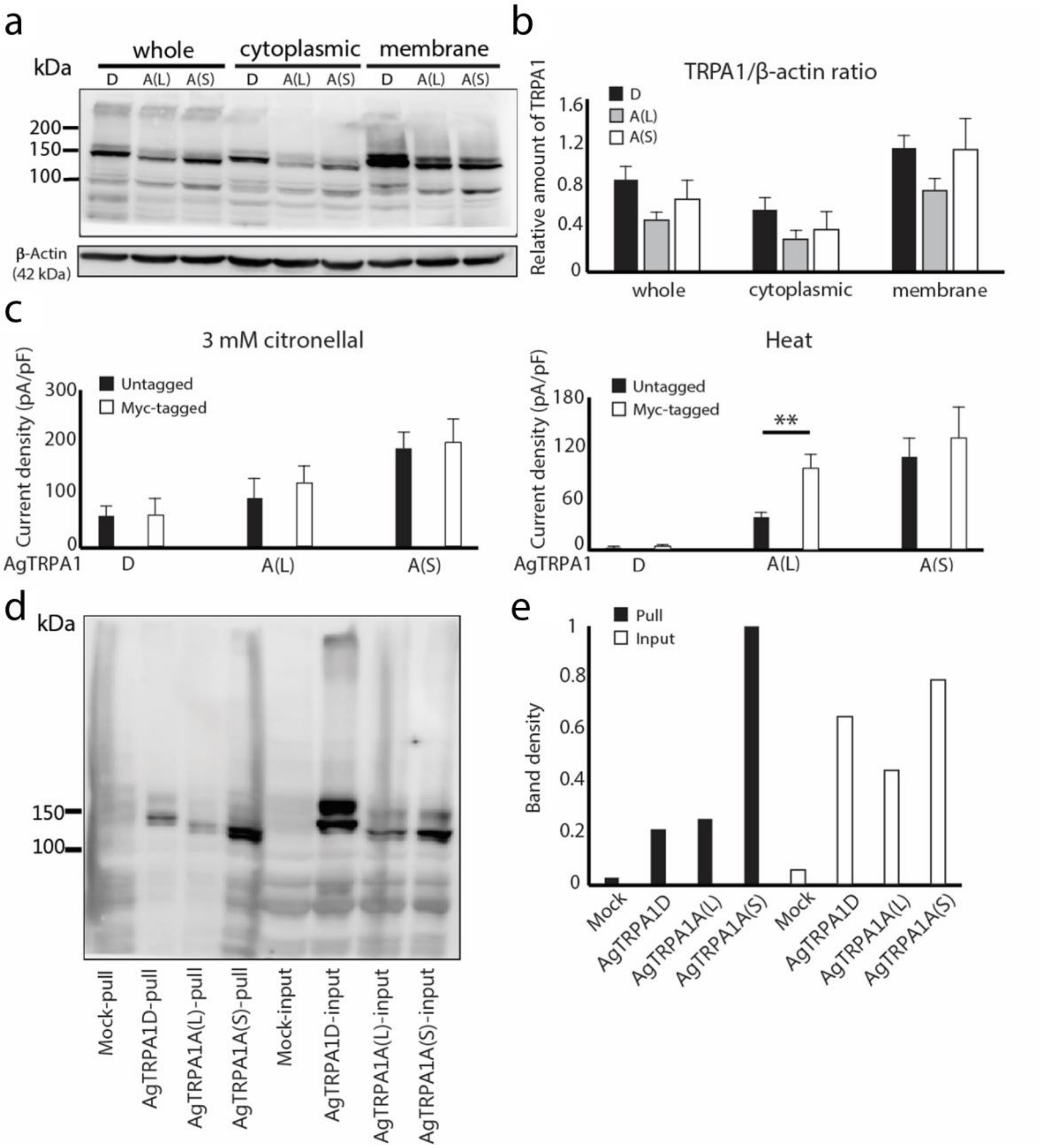

a
whole
cytoplasmic
membrane
kDa
D A(L) A(S) D A(L) A(S) D A(L) A(S)
200
150
100
β-Actin (42 kDa)
b
TRPA1/β-actin ratio
Relative amount of TRPA1
D
A(L)
A(S)
whole
cytoplasmic
membrane
c
3 mM citronellal
Heat
Current density (pA/pF)
Untagged
Myc-tagged
**
AgTRPA1
D
A(L)
A(S)
d
kDa
150
100
Mock-pull
AgTRPA1D-pull
AgTRPA1A(L)-pull
AgTRPA1A(S)-pull
Mock-input
AgTRPA1D-input
AgTRPA1A(L)-input
AgTRPA1A(S)-input
e
Pull
Input
Band density
Mock
AgTRPA1D
AgTRPA1A(L)
AgTRPA1A(S)

Fig. S5

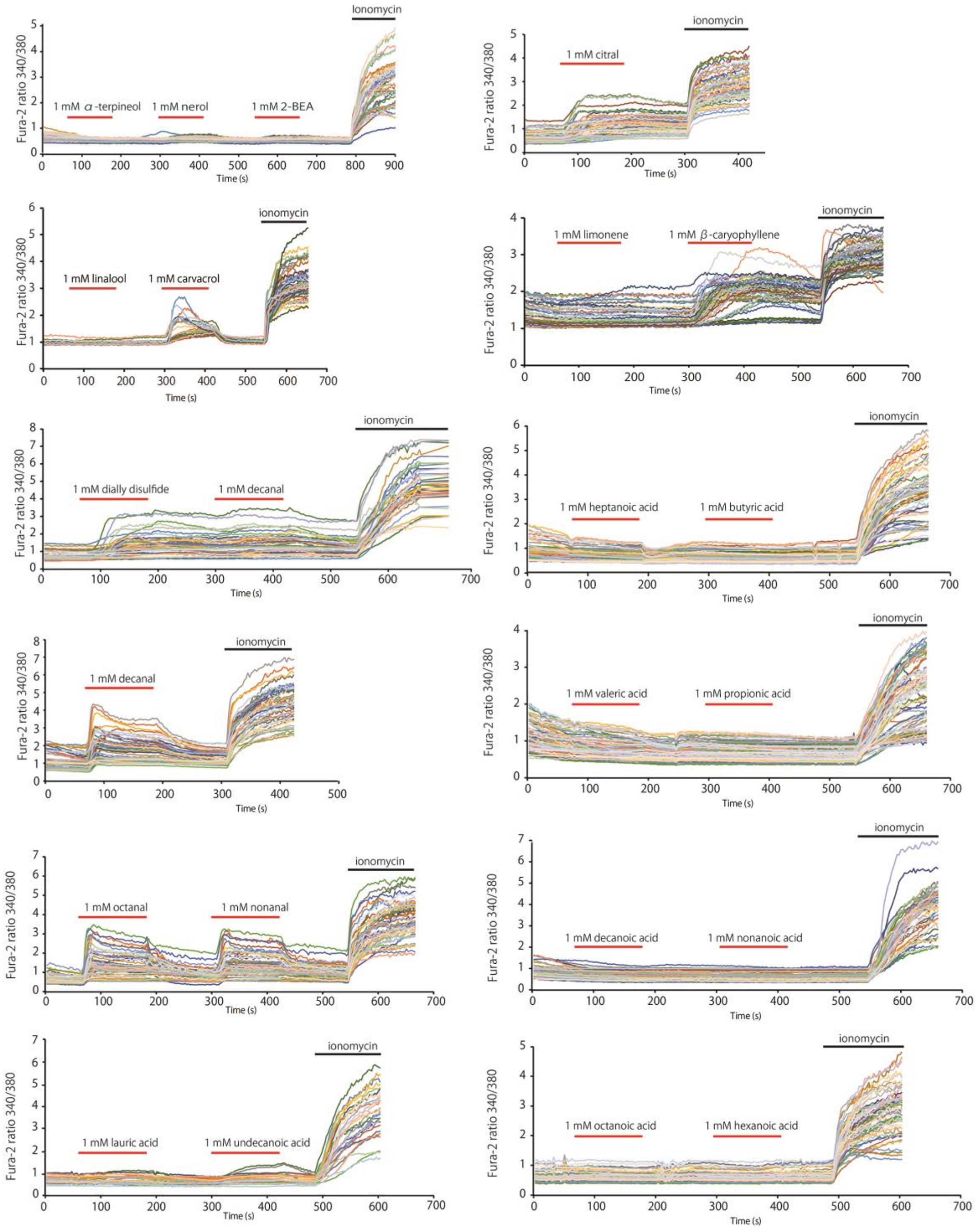

Ionomycin
1 mM α-terpineol
1 mM nerol
1 mM 2-BEA
ionomycin
1 mM citral
1 mM linalool
1 mM carvacrol
1 mM limonene
1 mM β-caryophyllene
1 mM dially disulfide
1 mM decanal
1 mM heptanoic acid
1 mM butyric acid
1 mM valeric acid
1 mM propionic acid
1 mM octanal
1 mM nonanal
1 mM decanoic acid
1 mM nonanoic acid
1 mM lauric acid
1 mM undecanoic acid
1 mM octanoic acid
1 mM hexanoic acid
Fura-2 ratio 340/380
Time (s)

Fig. S6

Supplementary Table S1

a

Species	PCR step	5' RACE primer sequences	3' RACE primer sequences
Anopheles stephensi	1st step	5'-GTGTACGGCCGAGTGCAGTG-3'	5'-TTCACTCGCATCTGCTGGACC-3'
	2nd step	5'-CTCCCGCGTGCATCCCTTCG-3'	5'-AAGCTGCTGCACAACTACATGG-3'
Aedes aegypti	1st step	5'-GGTGAGAATATTGATGCGGTTCC-3'	5'-TTCACTCGCATCTGCTGGACC-3'
	2nd step	5'-CGTCCTTTGCTATCCTTCACC-3'	5'-AAGCTGCTGCACAACTACATGG-3'
Culex pipiens pallens	1st step	5'-AGAAAGTCCATCTCGCCCAGCATC-3'	5'-GGAAAAGGGAACCTTCATCATCAACG-3'
	2nd step	5'-CATGACGACGTAGAGACCAACCTG-3'	5'-CGCAACGATGGAGAACAGACC-3'

b

Species	PCR step for variant *TrpA1C* or *TrpA1D*	Primer sequences	PCR step for variant *TrpA1A* or *TrpA1B*	Primer sequences
Anopheles stephensi	1st step	F 5'-TCAGTCGTTCATCAACAGCATC-3'	1st step	F 5'-CATCGAAGGTACAGCAGTAGCAG-3'
		R 5'-ACACACACGTACACGTACAAGCTC-3'		R 5'-ACACACACGTACACGTACAAGCTC-3'
	2nd step	F 5'-TCAGTCGTTCATCAACAGCATC-3'	2nd step	F 5'-CCCAAACCCATCAAGTGCAC-3'
		R 5'-TAAGCGGCCGCGTGGCTCCGCTACAGTGC-3'		R 5'-TAAGCGGCCGCGTGGCTCCGCTACAGTGC-3'
	3rd step	F 5'-CTTGTGTCCTCTGAACGATGC-3'		
		R 5'-TAAGCGGCCGCACAGTGCCATTCATTTTCC-3'		
Aedes aegypti	1st step	F 5'-CAATCAGACGAAATCCACCACTAGCC-3'	1st step	F 5'-CTACTGTAAACCGTCCATCG-3'
		R 5'-CAAAATTCGTTGTCCTGCTG-3'		R 5'-CAAAATTCGTTGTCCTGCTG-3'
	2nd step	F 5'-CTCGGATCCACCATGCCAACTCCGCTC-3'	2nd step	F 5'-CTCGGATCCACCATGTTACCAATTATGATG-3'
		R 5'-TAAGCGGCCGCCTACTTGCTAATCGACTTG-3'		R 5'-TAAGCGGCCGCCTACTTGCTAATCGACTTG-3'
Culex pipiens pallens	1st step	F 5'-CAACCCCCTACGGAAGAATCGCTAC-3'	1st step	F 5'-GTCCACGGTAACCATCGTCCATATCAG-3'
		R 5'-TCTCTCGCATTACTTGCTCATGGACTTG-3'		R 5'-TCTCTCGCATTACTTGCTCATGGACTTG-3'
	2nd step	F 5'-CTTGGATCCACCATGCCAACTCCGCTG-3'	2nd step	F 5'-CTCGGATCCACCATGATTAACCGGAAATCAATC-3'
		R 5'-TAAGCGGCCGCTTACTTGCTCATGGACTTG-3'		R 5'-TAAGCGGCCGCTTACTTGCTCATGGACTTG-3'
Anopheles gambiae	*TrpA1(A)*	R 5'-TTTGGATCCACCATGCCTACTCCGCTG-3'	*TrpA1(B)*	R 5'-TTAGGATCCACCATGTGGCGCAACTG-3'
	subcloning	R 5'-TAAGCGGCCGCTCATTTGCCAATAGATTTG-3'	subcloning	R 5'-TAAGCGGCCGCTCATTTGCCAATAGATTTG-3'

c

AgTrpA1 variants		Primer sequences
D	Add 2 Myc tags with double gly-gly-gly-ser linker	F 5'-CATGCCTACTCCGCTGTACTTAATTCATAG-3'
		R 5'-GAGCCGCCTCCGCTTCCGCCTCCCAAGTCCTCTTCA GAAATGAGCTTTTGCTCCATATCAAGCTTCAAGTCC-3'
A(L)		F 5'-CATGTTATCTGTTGCTATGTATACACGC-3'
		R 5'-GAGCCGCCTCCGCTTCCGCCTCCCAAGTCCTCTTCA GAAATGAGCTTTTGCTCCATATCAAGCTTCAAGTCC-3'
A(S)		F 5'-CATGTGGCGCAACTGTGCACAAC-3'
		R 5'-GAGCCGCCTCCGCTTCCGCCTCCCAAGTCCTCTTCA GAAATGAGCTTTTGCTCCATATCAAGCTTCAAGTCC-3'
